# Fetal cannabidiol (CBD) exposure alters thermal pain sensitivity, problem-solving, and prefrontal cortex excitability

**DOI:** 10.1038/s41380-023-02130-y

**Published:** 2023-07-11

**Authors:** Karli S. Swenson, Luis E. Gomez Wulschner, Victoria M. Hoelscher, Lillian Folts, Kamryn M. Korth, Won Chan Oh, Emily Anne Bates

**Affiliations:** 1 Section of Developmental Biology, Department of Pediatrics, University of Colorado Anschutz Medical Campus, Aurora, CO USA; 2 Department of Pharmacology, University of Colorado Anschutz Medical Campus, Aurora, CO USA

**Keywords:** Neuroscience, Psychiatric disorders, Physiology

## Abstract

Thousands of people suffer from nausea with pregnancy each year. Nausea can be alleviated with cannabidiol (CBD), a primary component of cannabis that is widely available. However, it is unknown how fetal CBD exposure affects embryonic development and postnatal outcomes. CBD binds and activates receptors that are expressed in the fetal brain and are important for brain development, including serotonin receptors (5HT_1A_), voltage-gated potassium (Kv)7 receptors, and the transient potential vanilloid 1 receptor (TRPV1). Excessive activation of each of these receptors can disrupt neurodevelopment. Here, we test the hypothesis that fetal CBD exposure in mice alters offspring neurodevelopment and postnatal behavior. We administered 50 mg/kg CBD in sunflower oil or sunflower oil alone to pregnant mice from embryonic day 5 through birth. We show that fetal CBD exposure sensitizes adult male offspring to thermal pain through TRPV1. We show that fetal CBD exposure decreases problem-solving behaviors in female CBD-exposed offspring. We demonstrate that fetal CBD exposure increases the minimum current required to elicit action potentials and decreases the number of action potentials in female offspring layer 2/3 prefrontal cortex (PFC) pyramidal neurons. Fetal CBD exposure reduces the amplitude of glutamate uncaging-evoked excitatory post-synaptic currents, consistent with CBD-exposed female problem-solving behavior deficits. Combined, these data show that fetal CBD exposure disrupts neurodevelopment and postnatal behavior in a sex specific manner.

## Introduction

The nausea of morning sickness is debilitating for thousands of pregnant patients each year [1]. Pregnant people are drawn to use cannabis for its anti-emetic, or anti-nausea, properties because they believe it to be safe [2]. Cannabis has two primary component parts, cannabidiol (CBD) and tetrahydrocannabinol (THC), along with minor cannabinoids and terpenes. Although research quantifying CBD consumption in a pregnant population is not yet published, THC metabolites were detected in cord blood samples from twenty-two percent of pregnant people [3], suggesting CBD consumption in the same group [4]. CBD is an effective anti-emetic medication [5–7], but does not induce the psychoactive properties of THC. CBD has become widely available since it was removed from schedule 1 drug classification in 2018 [8]. In addition to the people who consume CBD as a component of cannabis, many pregnant patients consume CBD alone [6].

CBD diffuses through maternal-placental-fetal circulation [9]. Lipophilic CBD accumulates in the fetal brain, liver, and gastrointestinal tract [9]. CBD binds and activates receptors important for fetal brain development including the 5HT_1A_ serotonin receptor, heat-activated transient potential vanilloid receptor one (TRPV1) calcium channels [10], and voltage-gated Kv7 receptor potassium channel [11–13], among others.

Excessive TRPV1 activation confers neural tube defects akin to those induced by maternal fever [14], suggesting that increased activation of TRPV1 affects developmental processes. Excessive activation of TRPV1 during gestation induces anxiety-like behavior in mice [15]. TRPV1 mediates excitatory innervation in the hippocampus and is required for plasticity [16]. In the fetal and postnatal brain, TRPV1 is expressed in limbic regions which mediate behavioral responses to stimuli [17–19]. Fetal cannabis exposure is associated with increased anxiety in humans, but whether CBD contributes to this association is unknown [20]. Because CBD activates TRPV1 and TRPV2, we hypothesize that fetal CBD exposure could disrupt brain development and affect thermal sensitivity, memory, and anxiety-like behaviors.

Excessive fetal serotonin signaling harms neuronal development [22, 23]. During fetal development, serotonin receptors are highly expressed in the prefrontal cortex (PFC), a region of the brain that mediates cognition [21]. Overexpression of 5HT_1A_ during mouse fetal and early postnatal development decreases adult anxiety-like behaviors and decreases spatial learning in mice [22]. Excessive activation of 5HT_1A_ during fetal development decreases neurogenesis, decreases neuron network complexity, alters neuron refinement, delays sensory-evoked potentials, decreases sensory evoked firing, and decreases amplitude of sensory evoked potentials [23]. Depletion of tryptophan, the molecular precursor to serotonin, impairs cognition in humans and mice [24, 25]. 5HT receptors are expressed in the fetal hippocampus and cortex in humans and mice [26] [27]. CBD activates 5HT_1A_, which mediates several important neurodevelopmental processes. We hypothesized that fetal CBD exposure could disrupt similar processes to reduce problem-solving behaviors or alter memory and anxiety-like behaviors.

CBD binds and activates Kv7.2/3, which are expressed in the brain throughout embryonic and postnatal development [28]. Kv7.2/3 gain-of-function mutations are associated with increased rates of human intellectual disability and epileptic encephalopathy [29]. Kv7.2/3 agonism decreases relative refractory periods and increases post-conditioned super-excitability of neurons in cultured human myelinated axons [30]. Alterations in fetal Kv7.2/3 activity cause cognitive impairment and memory deficits in mice [31]. CBD activates Kv7 receptors which are expressed in the fetal brain and excessive activation is associated with negative neurodevelopmental effects. We hypothesize that fetal CBD exposure could excessively activate Kv7.2/3 to disrupt brain development and postnatal behavior.

CBD is commonly consumed orally [32]. We administered high dose CBD dissolved in sunflower oil or sunflower oil alone to C57BL6 female dams daily from E5 through birth. Offspring were subjected to a battery of behavioral testing to determine if fetal CBD exposure alters postnatal behavior. We show that CBD-exposed male offspring are sensitized to thermal pain in a TRPV1-dependent manner. We found fetal CBD exposure did not affect offspring anxiety-like behaviors, spatial memory, or compulsivity. We show fetal CBD exposure reduces problem-solving behaviors in female mice. We demonstrate that fetal CBD exposure reduces excitability of pyramidal neurons in the prefrontal cortex in postnatal (P)14-21 female mice. Female CBD-exposed offspring require larger currents and more depolarized voltage to elicit action potentials and elicit fewer action potentials at a given current. Fetal CBD exposure reduced the amplitude of glutamate uncaging-evoked excitatory post-synaptic currents in female prefrontal cortical slices. CBD metabolites were not retained in pup plasma by P8 suggesting that fetal CBD exposure changes fetal neuronal physiology and neurodevelopment to impact postnatal behavior.

## Results

### CBD exposure does not alter pregnancy length, gestational weight gain, litter size, or sex of offspring

**Fig. 1:**
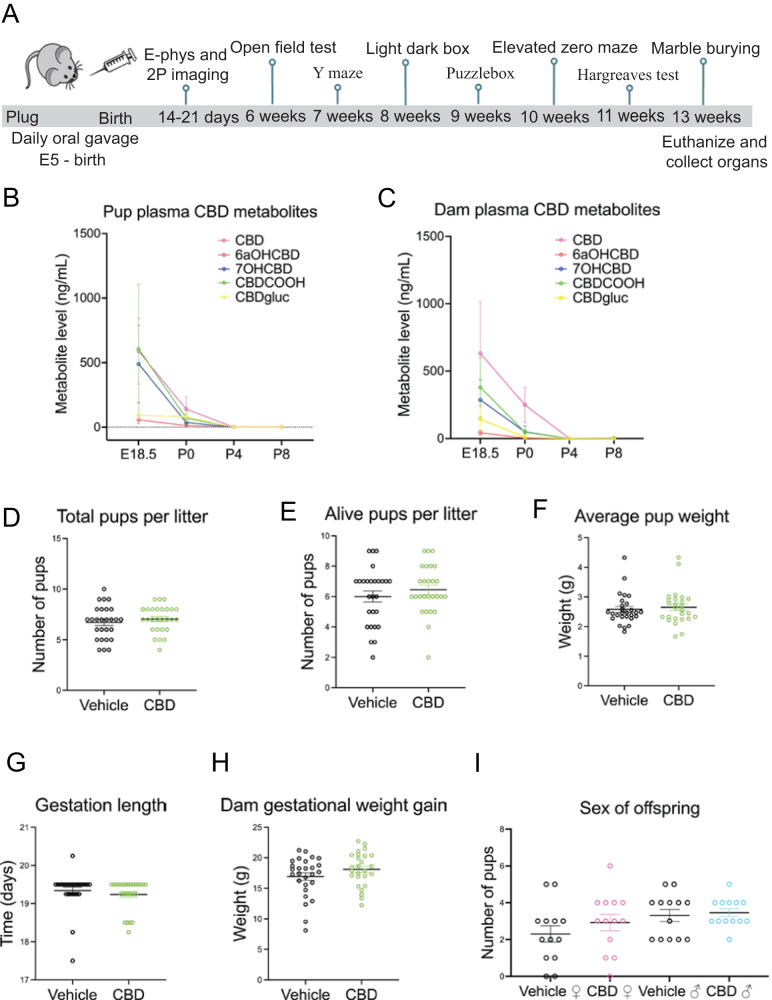
Dosing schematic, validation of CBD metabolites and litter factors. A timeline shows CBD administration and age of offspring when tests were performed (**A**). A graph shows CBD and CBD metabolites in the dam blood plasma from E18.5, P0, P4, and P8 (**B**) and pooled pup litter plasma from each group (**C**) from E18.5, P0, P4, and P8. Graphs show gestational CBD consumption does not alter total pups per litter (**D**), alive pups per litter (**E**), average pup weight (**F**), gestation length (**G**), gestation weight gain (**H**), or sex of offspring (**I**) from 27 vehicle administered dams and 26 CBD administered dams. Error bars represent S.E.M. No measures were significantly different based on treatment (*p* > 0.1 by t-test). Mean values, SEM, and *p* values by t-test are reported in Tables 1 and 2.

We administered 50 mg/kg CBD dissolved in sunflower oil or sunflower oil alone (vehicle) via oral gavage daily from E5 through birth to C57BL6J or *TRPV1*^*KO/KO*^ female mice (Fig. 1A). CBD is metabolized into 6a-hydroxy cannabidiol, 7-hydroxy cannabidiol, carboxy-cannabidiol, and cannabidiol glucuronide and each crosses the placenta [9]. We used liquid chromatography tandem mass spectrometry to quantify CBD and its metabolites in dam and pup plasma from 2 h after dosing on E18.5 and postnatal day (P) 0, P4, P8, and P12. CBD and its metabolites were detected in dam and pup plasma at E18.5, P0, and P4 (Fig. 1B, Table 1). Pups and dams had cleared the CBD and its metabolites by P8 (Fig. 1B, Table 1), suggesting that any behavioral or physiological differences between CBD and vehicle-exposed offspring subsequently measured are due to changes in development rather than acute effects of CBD. To determine how CBD consumption during pregnancy affects maternal factors, we quantified number of pups per litter, pup survival, average pup weight, pup sex ratios, gestation length and dam gestational weight gain in vehicle and CBD dosed dams and their litters. We found that CBD exposure did not alter any of these factors (Fig. 1D–I, Table 2). We conclude that CBD consumption during pregnancy, from E5 through birth, does not induce detectable changes in these maternal factors or litter composition compared to vehicle.

**Table 1:** CBD and CBD metabolites are found it dam and combined littermate plasma in E18.5 and P0.

	CBD	6aOHCBD	7OHCBD	CBDCOOH	CBDgluc
E18.5 pup	588.6 ± 253.3	58.3 ± 29.2	490.1 ± 298.6	606.2 ± 26.8	92.5 ± 26.8
P0 pup	139.7 ± 98.4	13.5 ± 4.2	37.6 ± 10.3	70.5 ± 31.8	82.5 ± 33.3
P4 pup	0.8 ± 0.5	2.5 ± 0.2	0 ± 0	0 ± 0	0 ± 0
E18.5 dam	630.9 ± 383.7	43.4 ± 18.8	286.6 ± 150.1	380.0 ± 220.5	143.4 ± 85.4
P0 dam	250.2 ± 130.5	4.0 ± 4.0	50.6 ± 42.3	48.2 ± 44.2	10.3 ± 6.9
P4 dam	2.1 ± 1.5	0 ± 0	0 ± 0	0 ± 0	0 ± 0

**Table 2:** Gestational oral consumption of CBD does not affect maternal or litter factors.

Treatment	Gestational weight gain	Gestation length	Pups per litter	Pups alive at weaning	Sex of offspring
CBD (*n* = 27 litters)	18.11 ± 0.53 grams	19.27 ± 0.073 days	7.04 ± 0.25	6.44 ± 0.31	2.92 ± 0.44 female 3.46 ± 0.22 male
Vehicle (26 litters)	16.93 ± 0.63 grams	19.3 ± 0.092 days	6.70 ± 0.33	6.00 ± 0.36	2.31 ± 0.44 female 3.31 ± 0.33 male
P (t-test)	0.413	0.64	0.61	0.36	0.70

### Fetal CBD exposure increases thermal pain sensitivity in male, but not female, offspring

**Fig. 2:**
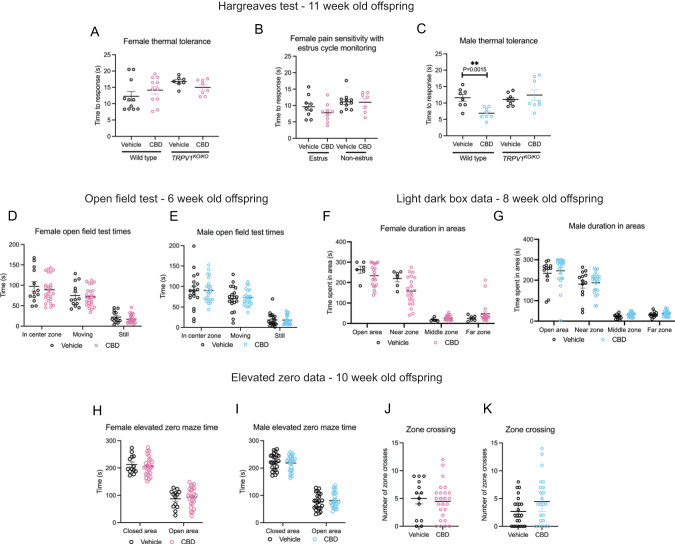
Fetal CBD exposure increases thermal sensitivity in male mice, but not female mice. Fetal CBD exposure does not affect latency to response to thermal stimulus in the Hargreaves test in wildtype or *TRPV1*^*KO/KO*^ female mice (**A**), and over the estrus cycle (**B**). Fetal CBD exposure decreases latency to response in wild-type CBD-exposed male mice (11.58 ± 0.64 s for vehicle-exposed vs 6.87 ± 3.27 s, *P* = 4.993E−8, t-test), but does not affect latency response in *TRPV1*^*KO/KO*^ mice (11.089 ± 0.649 s vehicle-exposed, vs. 12.429 ± 1.610 s CBD-exposed *P* = 0.453, t-test) (**C**). Graphs show fetal CBD exposure does not affect time in the center zone, time moving, or time still in the Open field test in 6-week-old female (**D**), or male offspring (**E**). Graphs show fetal CBD exposure does not affect time in open area, near zone or far zone in the Light Dark Box in 8-week-old female (**F**) or male (**G**) offspring. The elevated zero maze shows that fetal CBD exposure does not affect time in closed or open areas in 10-week-old female (**H**), and male (**I**) offspring. Fetal CBD exposure did not affect zone crossings in female (**J**), nor male offspring (**K**) in the elevated zero maze. Error bars represent the S.E.M. All mean values of measurements, S.E.M., and *p* values from t-tests are reported in Table 2.

We tested the hypothesis that fetal CBD exposure would excessively activate TRPV1 channels and alter the development of thermal pain circuits using the Hargreaves test in wild-type and *TRPV1*^*KO/KO*^ vehicle or CBD-exposed offspring. The Hargreaves test measures the latency to response to a thermal stimulus [33]. Fetal CBD exposure did not affect female sensitivity to thermal pain (12.25 ± 1.46 s, *N* = 11 vehicle-exposed versus 14.14 ± 1.21 s, *N* = 11 CBD-exposed, *P* = 0.331, t-test). *TRPV1*^*KO/KO*^ vehicle and CBD-exposed females were similarly sensitive to thermal stimuli (16.76 ± 0.60 s, *N* = 7 vehicle-exposed *TRPV1*^*KO/KO*^, 14.98 ± 0.82 s *N* = 8 CBD-exposed *TRPV1*^*KO/KO*^, *P* = 0.11, t-test. Figure 2A. Female pain tolerance varies with estrus cycle [34]. We repeated the Hargreaves test with female offspring controlling for estrus cycle stage and found no differences in thermal pain sensitivity based on fetal CBD exposure (Fig. 2B (9.62 ± 1.13 s *N* = 9 vehicle-exposed estrus compared to 7.79 ± 0.93 seconds, *N* = 9 CBD-exposed estrus females, *P* = 0.23, t-test), (11.11 ± 0.78 s *N* = 11 vehicle-exposed non-estrus females 10.99 ± 1.18 s *N* = 7 CBD-exposed non-estrus female mice, *P* = 0.93, t-test). CBD-exposed male offspring were significantly more sensitive to thermal pain than vehicle exposed controls (11.58 ± 0.64 seconds, *N* = 8 vehicle-exposed vs 6.87 ± 3.27 s, *N* = 8 CBD-exposed, *P* = 4.99E−8, t-test). CBD exposure did not impact thermal sensitivity in *TRPV1*^*KO/KO*^ male offspring (11.09 ± 0.65 s, *N* = 8 vehicle-exposed, vs. 12.43 ± 1.61 s, *N* = 8, *P* = 0.45, t-test, Fig. 2C) suggesting that CBD increases thermal pain sensitivity in males through exposure to a TRPV1 agonist. These data show that oral consumption of 50 mg/kg CBD during mouse pregnancy was sufficient to increase thermal pain sensitivity in male offspring.

### Fetal CBD exposure does not alter offspring anxiety-like behaviors or compulsivity

**Fig. 3:**
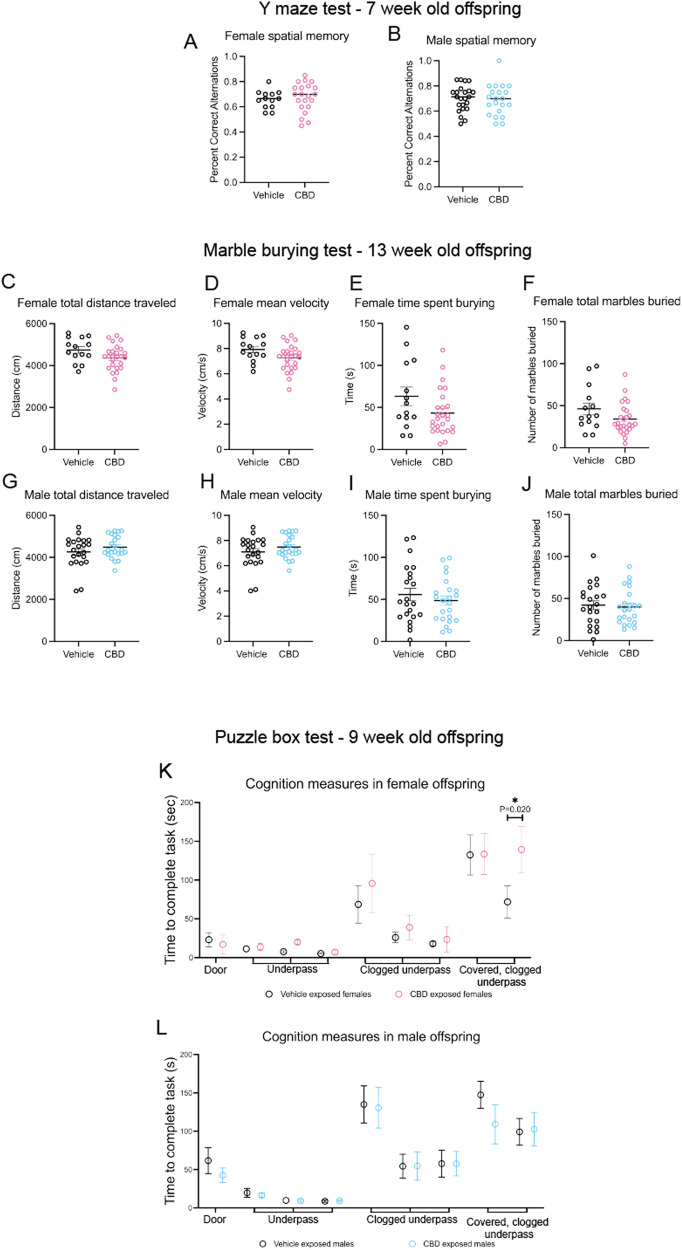
Fetal CBD exposure decreases female offspring problem-solving behaviors. Fetal CBD exposure does not affect female spatial memory (**A**) or male spatial memory (**B**) via the Y maze test. Graphs show fetal CBD exposure does not affect offspring compulsivity, including female total distance traveled (**C**), mean velocity (**D**), time spent burying (**E**) or marbles buried (**F**), nor male distance traveled (**G**), mean velocity (**H**), time spent burying (**I**) or total marbles buried (**J**), via the marble burying test. Graphs show fetal CBD exposure decreases female problem-solving at trial 9, (71.75 ± 20.71 s vehicle-exposed females, 139.42 ± 26.91 s CBD-exposed females, *N* = 12 each, *P* = 0.201, Wilcoxon rank sum test) (**K**), but not male problem-solving (**L**) via the puzzle box test.

Fetal TRPV1 activity mediates offspring anxiety-like behaviors in mice [15, 35]. Children exposed to whole cannabis in-utero have higher rates of anxiety and ADHD at puberty [20]. To determine if fetal CBD exposure affects offspring anxiety-like behaviors, we conducted the open field maze at six weeks, the light dark box at eight weeks, and the elevated zero maze test at ten weeks after birth (Fig. 2D–K, Table 3). We found no differences in anxiety-like behaviors by any measure in male or female offspring based on fetal CBD exposure (Fig. 2K, Supplementary Fig. 1, Table 3). We found significant differences in anxiety-like behaviors between *TRPV1*^*KO/KO*^ and wildtype mice, as previously characterized [35] (Supplementary Fig. 2). *TRPV1*^*KO/KO*^ mice also showed no differences in anxiety-like behaviors measures based on CBD exposure alone (Supplementary Fig. 2). To determine the effect of fetal CBD exposure on offspring compulsivity, we conducted the Marble Burying Test with mice exposed to vehicle or CBD during gestation. We found a trend towards CBD-exposed females spending less time burying marbles, but no differences in any measures for male offspring compulsivity based on CBD exposure (Fig. 3B–E, Table 4). These data show that neither anxiety-like behaviors nor compulsivity are significantly affected by fetal CBD exposure in mice (Fig. 3B–E, Tables 3 and 4).

**Table 3:** Fetal CBD exposure does not impact anxiety-like behaviors in male or female mice.

Test	Measure	Female vehicle			Female CBD			Female effect size	Female vehicle to female CBD	Distrobution	Test used
		*N* pups	*N* litters	Value ± SEM	*N* pups	*N* litters	Value ± SEM	Vehicle - CBD Mean	*P* value		
Open field test	Freuqnecy in center zone	13	5	49.93 ± 3.83	25	7	50.12 ± 2.29	-0.19	0.965	Normal	T-Test
Open field test	Velocity (cm/s)	13	5	11.34 ± 0.92	25	7	10.88 ± 0.51	0.46	0.525	Nonnormal	Wilcoxin rank sum test
Open field test	Total distance moved (cm)	13	5	932.47 ± 81.25	25	7	925.19 ± 50.00	7.28	0.936	Normal	T-Test
Open field test	Time in center zone (s)	13	5	90.20 ± 11.05	25	7	89.19 ± 6.36	1.01	0.933	Normal	T-Test
Open field test	Time moving (s)	13	5	70.46 ± 7.65	25	7	71.90 ± 4.62	−1.44	0.865	Normal	T-Test
Open field test	Time still (s)	13	5	19.72 ± 3.82	25	7	17.28 ± 2.10	2.44	0.225	Nonnormal	Wilcoxin rank sum test
Light dark box	Total distance moved in open area (cm)	6	3	740.45 ± 106.50	21	5	1033.55 ± 92.01	−293.1	0.071	Nonnormal	Wilcoxin rank sum test
Light dark box	Mean velocity (cm/s)	6	3	6.79 ± 0.30	21	5	6.83 ± 0.29	−0.04	0.572	Nonnormal	Wilcoxin rank sum test
Light dark box	Duration in open area (s)	6	3	263.09 ± 17.52	21	5	234.12 ± 11.89	28.97	0.225	Nonnormal	Wilcoxin rank sum test
Light dark box	Duration in near zone (s)	6	3	219.84 ± 16.48	21	5	159.26 ± 15.01	60.58	0.052	Normal	T-Test
Light dark box	Duration in middle zone (s)	6	3	17.34 ± 3.37	21	5	27.23 ± 2.54	−9.89	0.082	Normal	T-Test
Light dark box	Duration in far zone (s)	6	3	25. 28 ± 5.92	21	5	47.62 ± 11.22	−22.34	0.306	Nonnormal	Wilcoxin rank sum test
Light dark box	Latency to enter near zone (s)	6	3	1.49 ± 0.82	21	5	6.90 ± 4.79	−5.41	0.978	Nonnormal	Wilcoxin rank sum test
Light dark box	Latency to enter middle zone (s)	6	3	2.15 ± 1.17	21	5	18.85 ± 6.12	−16.7	0.112	Nonnormal	Wilcoxin rank sum test
Light dark box	Latency to enter far zone (s)	6	3	10.488 ± 5.497	21	5	20.30 ± 6.25	−9.812	0.428	Nonnormal	Wilcoxin rank sum test
Light dark box	Frequency in near zone	6	3	19.83 ± 3.84	21	5	27.33 ± 2.87	−7.5	0.207	Normal	T-Test
Light dark box	Frequency in middle zone	6	3	5.59 ± 2.28	21	5	20.33 ± 1.97	−14.74	0.184	Normal	T-Test
Light dark box	Frequency in far zone	6	3	7.00 ± 1.39	21	5	10.95 ± 1.55	−3.95	0.124	Nonnormal	Wilcoxin rank sum test
Elevated zero maze	Velocity (cm/s)	13	5	5.27 ± 0.36	25	7	5.25 ± 0.26	0.02	0.969	Normal	T-Test
Elevated zero maze	Frequency in light area	13	5	33.85 ± 3.35	25	7	31.68 ± 2.17	2.17	0.577	Normal	T-Test
Elevated zero maze	Total distance moved (cm)	13	5	1573.94 ± 108.78	25	7	1570.05 ± 77.47	3.89	0.976	Normal	T-Test
Elevated zero maze	Time in closed area (s)	13	5	212.67 ± 9.16	25	7	206.69 ± 6.93	5.98	0.977	Nonnormal.	Wilcoxin rank sum test
Elevated zero maze	Time in open area (s)	13	5	87.43 ± 9.16	25	7	93.41 ± 6.93	−5.98	0.977	Nonnormal	Wilcoxin rank sum test
Elevated zero maze	Frequncy in closed area	13	5	34 ± 3.34	25	7	31.6 ± 2.14	2.4	0.534	Normal	T-Test

Test	Measure	Male vehicle			Male CBD			Male effect size	Male vehicle to male CBD	Distrobution	Test used
		*N* pups	*N* litters	Value ± SEM	*N* pups	*N* litters	Value ± SEM	Vehilce - CBD Mean	*P* value		
Open field test	Freuqnecy in center zone	19	6	49.26 ± 4.78	23	7	48.87 ± 2.88	0.39	0.942	Normal	T-Test
Open field test	Velocity (cm/s)	19	6	10.69 ± 0.63	23	7	10.71 ± 0.54	−0.02	0.978	Nonnormal	Wilcoxin rank sum test
Open field test	Total distance moved (cm)	19	6	913.97 ± 90.52	23	7	930.76 ± 53.62	−16.79	0.869	Normal	T-Test
Open field test	Time in center zone (s)	19	6	89.40 ± 10.05	23	7	90.76 ± 6.00	−1.36	0.904	Normal	T-Test
Open field test	Time moving (s)	19	6	70.76 ± 6.95	23	7	72.53 ± 4.07	−1.77	0.821	Normal	T-Test
Open field test	Time still (s)	19	6	18.63 ± 3.72	23	7	18.23 ± 2.35	0.4	0.551	Nonnormal	Wilcoxin rank sum test
Light dark box	Total distance moved in open area (cm)	13	4	854.22 ± 89.37	24	6	1191.09 ± 165.01	−336.87	0.105	Nonnormal	Wilcoxin rank sum test
Light dark box	Mean velocity (cm/s)	13	4	7.12 ± 0.23	24	6	6.99 ± 0.50	0.13	0.484	Nonnormal	Wilcoxin rank sum test
Light dark box	Duration in open area (s)	13	4	233.82 ± 18.18	24	6	246.27 ± 14.30	−12.45	0.239	Nonnormal	Wilcoxin rank sum test
Light dark box	Duration in near zone (s)	13	4	180.42 ± 19.22	24	6	179.64 ± 13.14	0.78	0.973	Normal	T-Test
Light dark box	Duration in middle zone (s)	13	4	21.65 ± 2.99	24	6	31.87 ± 2.38	−10.22	0.014	Normal	T-Test
Light dark box	Duration in far zone (s)	13	4	31.75 ± 3.49	24	6	37.65 ± 2.78	−5.9	0.156	Nonnormal	Wilcoxin rank sum test
Light dark box	Latency to enter near zone (s)	13	4	3.57 ± 2.82	24	6	3.61 ± 1.68	−0.04	0.766	Nonnormal	Wilcoxin rank sum test
Light dark box	Latency to enter middle zone (s)	13	4	12.98 ± 4.98	24	6	13.35 ± 4.37	−0.37	0.915	Nonnormal	Wilcoxin rank sum test
Light dark box	Latency to enter far zone (s)	13	4	16.19 ± 5.65	24	6	15.85 ± 4.60	0.34	0.965	Nonnormal	Wilcoxin rank sum test
Light dark box	Frequency in near zone	13	4	21.23 ± 2.52	24	6	24.79 ± 2.67	−3.56	0.391	Normal	T-Test
Light dark box	Frequency in middle zone	13	4	18.62 ± 1.95	24	6	22.25 ± 1.72	−3.63	0.196	Normal	T-Test
Light dark box	Frequency in far zone	13	4	8.85 ± 0.89	24	6	10.54 ± 0.87	−1.69	0.193	Nonnormal	Wilcoxin rank sum test
Elevated zero maze	Velocity (cm/s)	23	7	4.79 ± 0.26	24	7	4.92 ± 0.26	−0.13	0.733	Normal	T-Test
Elevated zero maze	Frequency in light area	23	7	27.43 ± 2.15	24	7	27.04 ± 2.29	0.39	0.901	Normal	T-Test
Elevated zero maze	Total distance moved (cm)	23	7	1437.06 ± 78.82	24	7	1474.54 ± 79.04	−37.48	0.739	Normal	T-Test
Elevated zero maze	Time in closed area (s)	23	7	222.98 ± 6.21	24	7	218.35 ± 5.76	4.63	0.395	Nonnormal.	Wilcoxin rank sum test
Elevated zero maze	Time in open area (s)	23	7	77.12 ± 6.22	24	7	81.75 ± 5.76	−4.63	0.395	Nonnormal	Wilcoxin rank sum test
Elevated zero maze	Frequncy in closed area	23	7	27.30 ± 2.18	24	7	26.92 ± 2.32	0.38	0.904	Normal	T-Test

**Table 4:** Fetal CBD exposure does not impact compulsivity-like behaviors in male or female mice.

Marble burying	Female vehicle			CBD female			Female effect size	Female vehicle to female CBD
	*N* pups	*N* litters	Mean ± SEM	*N* pups	N litters	Mean ± SEM	Vehicle mean - CBD mean	*P* value
Total distance traveled (cm)	14	5	4744.9 ± 156.5	26	7	4362.9 ± 120.7	382	0.065
Mean velocity (cm/s)	14	5	7.93 ± 0.25	26	7	7.27 ± 0.20	0.66	0.054
Time spent burying (s)	14	5	63.19 ± 11.03	26	7	43.18 ± 5.35	20.01	0.073
# individual marbles buried	14	5	8.0 ± 0.30	26	7	7.73 ± 0.25	0.27	0.504
# marbles reburied	14	5	38.14 ± 6.87	26	7	26.31 ± 3.49	11.83	0.095
Total # of marbles buried	14	5	46.14 ± 7.06	26	7	34.04 ± 3.63	12.1	0.098
Percent time spent burying	14	5	10.53 ± 1.84	26	7	7.20 ± 0.89	3.33	0.073

Marble burying	Male vehicle			Male CBD			Male effect size	Male vehicle to male CBD
	*N* pups	*N* litters	Mean ± SEM	*N* pups	*N* litters	Mean ± SEM	Vehicle mean - CBD mean	*P* value
Total distance traveled (cm)	22	7	4486.9 ± 112.6	23	7	4256.5 ± 160.8	230.4	0.244
Mean velocity (cm/s)	22	7	7.49 ± 0.19	23	7	7.09 ± 0.27	0.4	0.235
Time spent burying (s)	22	7	48.52 ± 5.45	23	7	55.70 ± 7.44	−7.18	0.438
# individual marbles buried	22	7	7.96 ± 0.30	23	7	7.64 ± 0.47	0.32	0.563
# marbles reburied	22	7	31.96 ± 4.19	23	7	34.45 ± 4.88	−2.49	0.699
Total # of marbles buried	22	7	39.91 ± 4.40	23	7	42.09 ± 5.23	−2.18	0.751
Percent time spent burying	22	7	8.09 ± 0.91	23	7	9.28 ± 1.24	−1.19	0.438

### Fetal CBD exposure does not alter offspring spatial memory

To determine how fetal CBD exposure impacts offspring spatial memory, we conducted the Y maze test. We found no effect of CBD exposure, sex, or genotype (WT or *TRPV1*^*KO/KO*^) on spatial memory in the percent of correct alternations within the Y maze (Fig. 3A, 0.656 ± 0.020 percent correct alternations *N* = 13 vehicle females, 0.68 ± 0.025 percent correct alternations *N* = 21 CBD females, *P* = 0.63, t-test. 0.70 ± 0.021 percent correct alternations *N* = 23 vehicle males, and 0.69 ± 0.027 percent correct alternations *N* = 21 CBD males, *P* = 0.699, t-test).

### Fetal CBD exposure decreases problem-solving behaviors in female offspring

To determine if fetal CBD exposure impacts problem-solving behaviors, mice exposed to CBD or vehicle in-utero underwent the puzzle box test. This test introduces each mouse to a light box and presents a progressively harder problem-solving challenge to reach a dark goal area [36]. Each mouse completes nine trials, with novel progressive challenges at trials two, five, and eight. Mice with sufficient problem-solving skills decrease their time to the goal area after secondary exposure to the obstacle [36]. Male CBD-exposed offspring reached the goal box at similar times compared to vehicle-exposed male offspring (Fig. 3F, Table 5). Female CBD-exposed offspring took significantly more time to reach the goal area in trial 9 compared to the vehicle-exposed female offspring ((71.75 ± 20.71 s vehicle-exposed females, 139.42 ± 26.91 s CBD-exposed females, *N* = 12 each, *P* = 0.02, Wilcoxon rank sum test), Fig. 3F, Table 5). These data show that fetal CBD exposure impairs problem-solving behaviors in female mice.

**Table 5:** Fetal CBD exposure impairs problem-solving in female, but not male offspring.

Puzzle Box Test	Measure	Female vehicle			Female CBD			Female effect size
		*N* pups	*N* litters	Mean latency (sec) ± SEM	*N* pups	*N* litters	Mean latency (sec) ± SEM	Vehicle - CBD Mean
Opendoor	Trial 1	12	5	23.08 ± 9.20	12	4	17.00 ± 5.90	6.08
Underpass	Trial 2	12	5	11.42 ± 3.05	12	4	13.83 ± 2.41	−2.41
Underpass	Trial 3	12	5	7.58 ± 1.22	12	4	20.08 ± 12.29	−12.5
Underpass	Trial 4	12	5	5.18 ± 0.89	12	4	7.00 ± 1.45	−1.82
Clogged underpass	Trial 5	12	5	68.58 ± 24.31	12	4	95.75 ± 17.47	−27.17
Clogged underpass	Trial 6	12	5	26.00 ± 6.75	12	4	38.92 ± 9.95	−12.92
Clogged underpass	Trial 7	12	5	18.00 ± 2.67	12	4	23.50 ± 6.03	−5.5
Covered clogged underpass	Trial 8	12	5	132.50 ± 25.88	12	4	133.58 ± 35.93	97.65
Covered clogged underpass	Trial 9	12	5	71.75 ± 20.81	12	4	139.42 ± 26.91	−68.16

Puzzle Box Test	Measure	Male vehicle			Male CBD			Male effect size
		*N* pups	*N* litters	Mean latency (sec) ± SEM	*N* pups	*N* litters	Mean latency (sec) ± SEM	Vehicle - CBD mean
Opendoor	Trial 1	12	4	61.67 ± 16.88	12	4	42.58 ± 9.53	19.14
Underpass	Trial 2	12	4	19.67 ± 5.85	12	4	16.50 ± 2.75	3.17
Underpass	Trial 3	12	4	9.82 ± 2.85	12	4	9.18 ± 1.63	0.64
Underpass	Trial 4	12	4	8.75 ± 1.37	12	4	9.25 ± 0.84	−0.5
Clogged underpass	Trial 5	12	4	134.92 ± 24.15	12	4	130.58 ± 26.44	4.34
Clogged underpass	Trial 6	12	4	54.33 ± 15.73	12	4	54.67 ± 18.58	−0.34
Clogged underpass	Trial 7	12	4	57.75 ± 17.49	12	4	57.67 ± 15.95	0.08
Covered clogged underpass	Trial 8	12	4	147.42 ± 17.61	12	4	109.08 ± 25.59	38.34
Covered clogged underpass	Trial 9	12	4	99.25 ± 17.41	12	4	102.67 ± 21.86	−3.42

### Fetal CBD exposure decreases excitability of PFC L2/3 pyramidal neurons in a sex specific manner

**Fig. 4:**
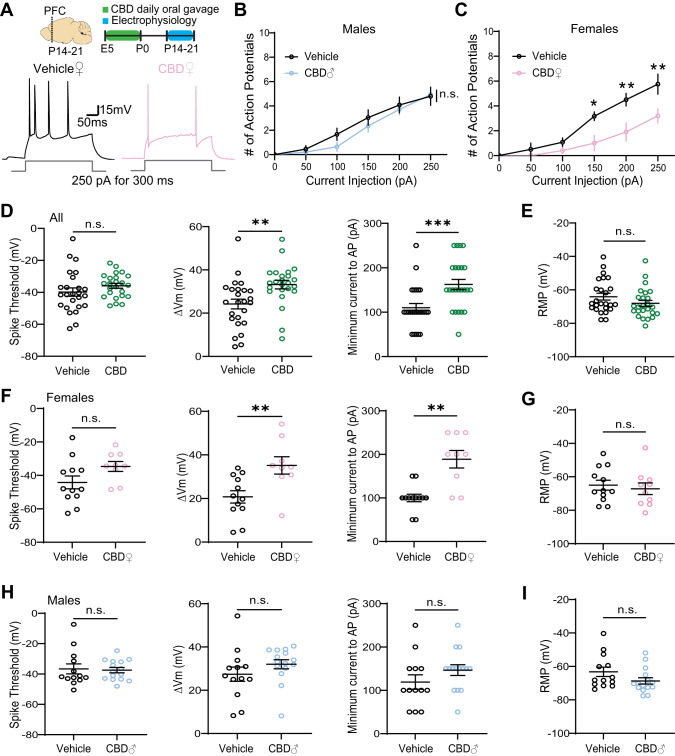
Fetal CBD exposure decreases excitability of PFC layer 2/3 pyramidal neurons in a sex specific manner. **A** Experimental timeline and representative traces of a stimulus train elicited by 250pA current injection for 300 ms. **B**, **C** Fetal CBD exposure decreased the intrinsic excitability of layer 2/3 pyramidal neurons in P14 females but not males (vehicle females: *n* = 6 cells, 1 mouse; vehicle males: *n* = 12 cells, 2 mice; CBD females: *n* = 5 cells, 1 mouse; CBD males: *n* = 14 cells, 2 mice; female treatment effect: *P* < 0.0001; Sidak’s multiple comparison, *<0.05, **<0.01). **D** Fetal CBD exposure did not alter spike thresholds of P14-21 mice (left, vehicle: −39.84 ± 2.613 mV, *n* = 25 cells, 4 mice; CBD: −35.92 ± 1.523 mV, *n* = 24 cells, 4 mice; Welch’s t-test, *P* = 0.2018). Fetal CBD exposure significantly increased membrane potential change (middle, vehicle: 24.23 ± 2.251 mV; CBD: 33.21 ± 1.963 mV; two-tailed t-test, *P* = 0.0043) and minimum currents (right, vehicle: 110 ± 9.574 pA; CBD: 162.5 ± 11.36; Mann–Whitney, *P* = 0.0007) required to evoke action potentials. **E** Resting membrane potential of P14-21 mice remained unchanged following fetal CBD exposure (vehicle: −64.07 ± 2.008 mV, *n* = 25 cells, 4 mice; CBD: −68.06 ± 1.773 mV, *n* = 25 cells, 4 mice; Mann–Whitney test, *P* = 0.1261). **F** The effect of fetal CBD exposure on changes of membrane potential (vehicle: 20.76 ± 2.826 mV, *n* = 12 cells, 2 mice; CBD: 35.19 ± 3.963, *n* = 10 cells, 2 mice; two-tailed t-test, *P* = 0.0066) and minimum current for action potential firing stemmed from females (vehicle: 100 ± 8.704 pA, *n* = 12 cells, 2 mice; CBD: 188.9 ± 20.03 pA, *n* = 10 cells, 2 mice; Welch’s t-test, *P* = 0.0018). **G** Resting membrane potential was unchanged in females following fetal CBD exposure (vehicle: −65.97 ± 2.966 mV, *n* = 12 cells, 2 mice; CBD: −67.16 ± 3.506 mV, *n* = 10 cells, 2 mice; two-tailed t-test, *P* = 0.6370). **H**, **I** Male mice showed no significant differences in spike threshold (vehicle: −35.81 ± 3.321 mV, *n* = 13 cells, 2 mice; CBD: −36.65 ± 1.725 pA, *n* = 15 cells, 2 mice; Mann–Whitney test, 0.7856), change in membrane potential (vehicle: 27.43 ± 3.309 mV; CBD: 32.02 ± 2.115 mV; Mann–Whitney test, *P* = 0.0648),and minimum currents for action potential spikes (vehicle: 119.2 ± 16.54 pA; CBD: 146.7 ± 12.41 pA; two-tailed t-test, *P* = 0.1893), or resting membrane potential (vehicle: −63.24 ± 2.820 mV; CBD: −68.67 ± 1.910, Mann–Whitney test, *P* = 0.0977). *P < 0.05, **P < 0.01, ***P < 0.001; error bars represent SEM. n.s. not significant.

We explored neural mechanisms that transduce fetal CBD exposure into decreased problem-solving behaviors in female mice (Fig. 4) with ex vivo electrophysiological recordings. We measured the intrinsic membrane properties of layer 2/3 pyramidal neurons in acute PFC slices from the CBD and vehicle-exposed male and female offspring. Fetal CBD-exposed female mice showed significantly decreased excitability (treatment effect, *p* < 0.0001, two-way ANOVA), while CBD-exposed male pups were comparable to age, sex-matched vehicle treated controls (treatment effect, *p* = 0.1711, two-way ANOVA; Fig. 4A–C). Combining sexes, fetal CBD exposure did not change the spike threshold (Vehicle: −39.84 ± 2.613 mV; CBD: −35.92 ± 1.523 mV; *p* = 0.2018) (Fig. 4D). However, we observed a significant increase in membrane potentials (Vehicle: 24.23 ± 2.251 mV; CBD: 33.21 ± 1.963 mV; *p* = 0.0043) and minimum currents required to trigger action potentials (Vehicle: 110 ± 9.574 pA; CBD: 162.5 ± 11.36; *p* = 0.0007) in pooled CBD-exposed mice (Fig. 4D). We found that these differences stem from alterations in the intrinsic properties of female (Fig. 4F), but not male mice (Fig. 4H), without affecting resting membrane potentials (Vehicle: −64.07 ± 2.008 mV; CBD: −68.06 ± 1.773 mV; p = 0.1261) (Fig. 4E, G, I). Together, these data demonstrate CBD-mediated and sex specific decrease in neuronal excitability of PFC layer 2/3 pyramidal neurons.

### Fetal CBD exposure affects excitatory synapse development in the PFC in a sex specific manner

**Fig. 5:**
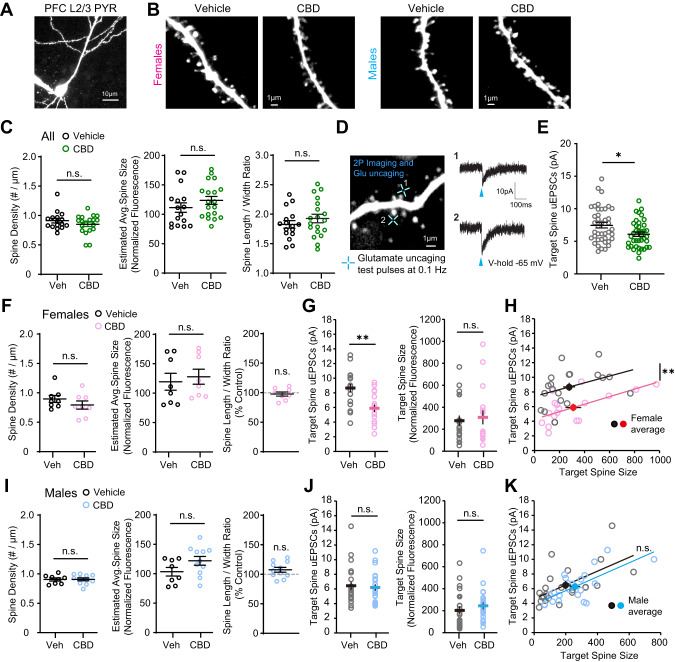
Fetal CBD exposure decreases synaptic strength of layer 2/3 pyramidal neurons in PFC of female mice. **A**, **B** Two-photon images of a whole-cell PFC layer 2/3 pyramidal neuron and dendritic segments from CBD and vehicle treated female and male mice at P14-22. **C** Fetal CBD exposure has no effect on spine structure for combined male and female mice (vehicle: *n* = 70 dendrites, 16 cells, 4 mice; CBD *n* = 84 dendrites, 19 cells, 4 mice). **D** A two-photon image from a dendritic segment of PFC layer 2/3 pyramidal neuron and two-photon glutamate uncaging evoked EPSC (uEPSC) traces (average of 5–8 test pulses) recorded by whole-cell voltage-clamp recording (blue crosses indicate glutamate uncaging timepoint). **E** uEPSC amplitudes are significantly decreased in CBD-exposed offspring (vehicle: 7.49 ± 0.42 pA; CBD: 6.09 ± 0.33 pA; *P* = 0.0116, two-tailed t-test) (vehicle: *n* = 41 spines, 14 cells, 3 mice; CBD *n* = 39 spines, 13 cells, 3 mice). **F** In female offspring, fetal CBD exposure has no effect on spine density, average spine size, or spine length/width ratio. (vehicle: *n* = 35 dendrites, 8 cells, 2 mice; CBD *n* = 36 dendrites, 8 cells, 2 mice). **G** uEPSCs recorded from similar sizes of target spines are significantly smaller in fetal CBD-exposed female offspring (vehicle: 8.66 ± 0.55 pA; CBD: 5.89 ± 0.51; *P* = 0.0009, two-tailed t-test) (vehicle: *n* = 19 spines, 7 cells, 1 mouse; CBD *n* = 17 spines, 6 cells, 1 mouse). **H** Scatter plots showing significantly smaller uEPSCs in fetal CBD-exposed mice. **I** In male offspring, fetal CBD exposure had no effect on spine density, average spine size, or spine length/width ratio. (vehicle: *n* = 35 dendrites, 8 cells, 2 mice; CBD *n* = 48 dendrites, 11 cells, 2 mice). **J** In male offspring, CBD has no effect on uEPSCs (vehicle: *n* = 22 spines, 7 cells, 2 mice; CBD *n* = 22 spines, 7 cells, 2 mice). **K** Scatter plots showing comparable uEPSCs between fetal CBD-exposed and control mice. *P < 0.05, **P < 0.01; ***P < 0.001; error bars represent SEM. n.s. not significant.

We investigated whether fetal CBD exposure changes structure and function of excitatory spine synapses on layer 2/3 pyramidal neurons in the PFC because excitatory synapse development is regulated by neuronal activity [37–39]. We examined spine density (Fig. 5A, B) and function (Fig. 5A, D) in acute PFC slices of CBD and vehicle-exposed mice using two-photon microscopy and simultaneous whole-cell patch clamp recordings and two-photon glutamate uncaging [40]. We found that spine density (Vehicle: 0.91 ± 0.04 #/µm; CBD: 0.85 ± 0.04 #/µm; *p* = 0.2513), size (Vehicle: 111.4 ± 8.07; CBD: 123.7 ± 6.76; *p* = 0.2479), and morphology (Vehicle: 1.83 ± 0.067; CBD: 1.93 ± 0.07; *p* = 0.3156) were unaffected by fetal CBD exposure (Fig. 5C). No sex specific changes were observed (Female, Vehicle: 0.90 ± 0.06 #/µm, CBD: 0.79 ± 0.07 #/µm, *p* = 0.2813; Male, Vehicle: 0.906 ± 0.03 #/µm; CBD: 0.907 ± 0.02 #/µm; *p* = 0.9750) (Fig. 5F, I). However, uncaging-evoked alpha-amino 3-hydroxy-5-methyl-4 isoxazole propionic acid receptor (AMPAR) currents (uEPSCs) were significantly decreased on CBD-exposed groups (Fig. 5D, E) compared to age-matched vehicle treated controls (Vehicle: 7.49 ± 0.42 pA; CBD: 6.09 ± 0.33 pA; *p* = 0.011). We found this effect was female specific. uEPSCs were significantly smaller in fetal CBD-exposed female offspring (Vehicle: 8.66 ± 0.55 pA; CBD: 5.89 ± 0.51; *p* = 0.0009) (Fig. 5G, H) with no effect on uEPSC amplitudes from CBD male mice (Vehicle: 6.48 ± 0.56 pA; CBD: 6.24 ± 0.45 pA; *p* = 0.898) (Fig. 5J, K). We targeted similar sizes of spines across groups as spine size and synaptic strength are strongly correlated [40]. These data show the female-specific effect of fetal CBD exposure on excitatory synapse development in the PFC.

## Discussion

CBD is easily accessible in many countries and helps with nausea, the most common adverse symptom of pregnancy. Our data demonstrate that fetal CBD exposure sensitizes males to thermal pain, decreases females problem-solving behaviors, and reduces excitability of prefrontal cortical pyramidal neurons from female mice. These results show CBD consumption during pregnancy can adversely affect fetal neurodevelopment in mice.

### Fetal CBD exposure induces thermal pain sensitivity in male offspring

Our data demonstrate fetal CBD exposure increases thermal pain sensitivity in 11-week-old male offspring. CBD metabolites are not detected in pups after P8 suggesting that fetal CBD exposure alters thermal pain sensing circuits during development. This effect was dependent on the TRPV1 receptor. TRPV1 receptors are activated by heat (40–45 °C) [41] and are bound and activated by CBD [42]. In a developmental context, altering the development of thermal pain circuits may have negative consequences on behavior long term. Persistent increased thermal pain sensitivity could increase susceptibility to chronic pain and may lay the groundwork for the use of pain-relieving medications like opioids. Fetal CBD exposure does not impact thermal sensitivity in *TRPV1*^*KO/KO*^ mice, suggesting that the effect of CBD on thermal pain circuit development depends on TRPV1 receptors. Because CBD is an agonist of TRPV1, these results suggest that exposure to an agonist of TRPV1 during fetal development can alter long-term thermal sensitivity. CBD-exposed female offspring responded to thermal stimuli similarly to vehicle-exposed controls. 17β-estradiol activation can downregulate TRPV1 activity in dorsal root ganglion sensory neurons [43] and female mice show different thermal pain sensitivity across the estrus cycle [44]. Thus, it is possible that estrogen could protect female offspring from excessive activation of TRPV1 by fetal exposure to CBD.

### Fetal CBD exposure does not impact offspring anxiety-like behavior or compulsivity

The prefrontal cortex (PFC) is a region of the brain that controls cognition, memory, anxiety, attention, and impulsivity [45]. The developing prefrontal cortex contains a multitude of receptors critical for normal development, including 5HT_1A_ serotonin receptors and Kv7 receptors that are bound and activated by CBD [46, 47]. We investigated multiple behaviors mediated by the prefrontal cortex, including anxiety-like behaviors and compulsivity. CBD-activated receptors, including TRPV1, are expressed in the hippocampus, a region of the brain that mediates memory [16]. We found that fetal CBD exposure did not impact offspring anxiety-like behaviors in wild-type or *TRPV1*^*KO/KO*^ mice by any measure in the open field test, the light dark box, or the elevated zero maze test. Previous studies administered 20 mg/kg CBD (Epidiolex) dissolved in honey via oral gavage from 14 days pre-conception through offspring weaning and found that the 12-week-old CBD-exposed female offspring buried more marbles in the marble burying test while male CBD-exposed offspring were no different than controls [48]. In contrast, we found that oral gavage of 50 mg/kg CBD from E5 through birth did not significantly affect anxiety-like behaviors measured by the open field, light/dark box, or elevated zero maze, nor measures of compulsivity on the marble burying test. This difference in effects of intrauterine CBD exposure on anxiety may be due to the dose or timing of exposures.

### Fetal CBD exposure decreases problem-solving behaviors in female offspring, but not male offspring

**Fig. 6:**
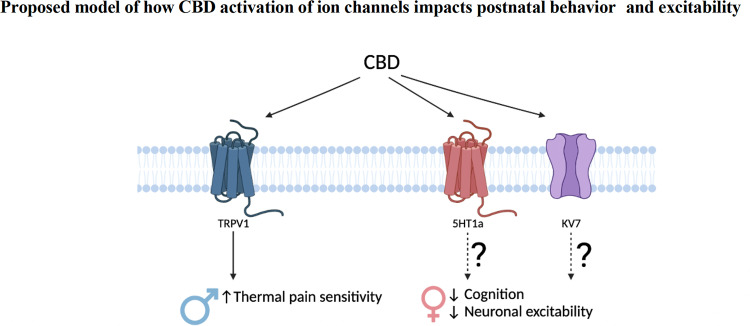
Model of effects of fetal CBD exposure. Fetal CBD exposure increases male thermal pain sensitivity, decreases female problem-solving behaviors, and alters female prefrontal cortex pyramidal neurons. CBD activates TRPV1, 5HT_1A_, and Kv7.2/3 receptors. TRPV1 regulates thermal pain sensitivity. 5HT1a and Kv7.2/3 have documented roles in neuronal excitability and cognitive function.

We show fetal CBD exposure reduces problem-solving behaviors in female offspring via the puzzle box test. Problem-solving behaviors are mediated by the prefrontal cortex [45]. We show that fetal CBD exposure reduces excitability of P14-P21 pyramidal neurons from the female prefrontal cortex. Fetal CBD exposure raised the required current to elicit an action potential, raised the required voltage to elicit an action potential, and decreased the number of action potentials elicited at set current in female, but not male, offspring (Fig. 4). It will be interesting to determine which CBD receptors are responsible for the long-term reduction in excitability of layer 2/3 pyramidal neurons in the prefrontal cortex and effects of fetal CBD exposure on cognitive function in female offspring. CBD activates 5HT_1A_ and Kv7.2/3, both of which mediate neuronal activity [8, 10] and are expressed in the fetal prefrontal cortex [43, 46] (Fig. 6). Future studies will focus on potential mechanisms by which fetal CBD exposure impairs neurodevelopment and the receptors that mediate these effects.

## Conclusions

Cannabis consumption during pregnancy is increasing [2]. Pregnant patients attempt to treat nausea symptoms with whole cannabis or CBD alone [2]. Clinical studies show fetal cannabis exposure is associated with adverse behavioral outcomes [49] though these clinical studies have not distinguished between cannabis component parts. Most cannabis products contain CBD [50]. There is an additional population who consume CBD alone because it is not psychoactive. Our work shows that a high dose fetal CBD exposure increases male offspring thermal pain sensitivity, reduces excitability of pyramidal neurons in the prefrontal cortex in female offspring, and decreases female offspring problem-solving behaviors. Our data fills a critical gap in the translational research focused on gestational cannabis consumption. This research is needed to inform public health messaging that CBD consumption during pregnancy can have adverse long-term neurodevelopmental outcomes. Further research is needed to determine sensitive periods of CBD exposure, the interaction of CBD with other cannabinoids like THC, and differential effects based on the route of administration.

## Methods

### Study design

C57Bl6J female mice were administered 50 mg/kg of CBD (98.7% pure powder, synthetic, National Institute of Drug Abuse) dissolved in sunflower oil or sunflower oil alone by oral gavage from E5 though birth. After first pass metabolism in the dam liver, oral gavage of 50 mg/kg is equivalent to 5 mg/kg intraperitoneal injection recommended by the national institutes of drugs of abuse (NIDA). Oral gavage replicates measured oral CBD consumption. Experimentalists and analysts were blind to exposure group through the entirety of behavioral testing. At 21 days old, offspring were weaned to standard chow, and cohoused with same-sex siblings. Our sample size includes 27 vehicle-exposed and 27 CBD-exposed dams, whose litter sizes vary (Fig. 1). Each behavior experiment was performed once per animal, within a two-week period to accommodate high volumes of offspring. Both exposure groups were represented in each individual trial.

### Animal protocols

Experiments were approved by the University of Colorado Anschutz Medical Campus Institutional Animal Care and Use Committee (protocols #139 and 721). Female C57BL6 mice (Strain #000664) and female *TRPV1*^*KO/KO*^ mice (Strain #003770) on a C57BL6 background (Jackson Laboratory, Maine). Dam weight was tracked each day starting on E0.5. Any mouse that had not gained appropriate weight by E14 was removed from the study.

### CBD administration

Cannabidiol (CBD) was obtained from NIDA with Drug Enforcement Administration (DEA) Schedule 1 Drug license (#RB0605026) approval. 500 mg of CBD was diluted in 40 ml sunflower oil and heated to 60 °C to make a 12.5 mg/ml concentration in an amber glass vial. Diluted CBD was evaluated for purity by the iC42 lab at the University of Colorado Anschutz Medical Campus. Consumption method (injection, oral consumption, inhalation) affects pharmacokinetic CBD breakdown. CBD is most commonly consumed topically or orally [32]. CBD clears more slowly with oral consumption than with IP injection [51]. The primary active metabolite of CBD is 7-OH-CBD reducing bioavailability of CBD to 10–13% of the initial dose [52]. We multiplied the standard research dose (5 mg/kg administered as i.p. injection) by ten to create a comparable oral dose of 50 mg CBD/kg body weight, well below the dose that would induce hepatotoxicity when administered via oral gavage over multiple days [53].

### Plasma metabolite concentration

Dam blood was collected via decapitation or cardiac puncture. On E18.5, dams were euthanized via isoflurane inhalation and secondary cervical dislocation. For all time points, blood was collected and stored in EDTA tubes (Microvette 100 KE3 Kent Scientific Corporation, item ID: MCVT100-EDTA). Blood was centrifuged at 3000 × *g* for 10 min at 4 °C to separate plasma. Plasma was stored in a clean EDTA tube at −80 °C until transfer to the iC42 Clinical Research and Development (Aurora, CO). CBD, 6a-hydroxy-CBD, 7-hydroxy-CBD, carboxy-CBD, and CBD glucuronide were quantified using high-performance liquid chromatography-tandem mass spectrometry (LC-MS/MS) as previously described [54]. The results included in the study sample batch met predefined acceptance criteria: the calibration range for 6a-hydroxy-CBD, 7-hydroxy-CBD and carboxy-CBD are 1.56–400 ng/mL, CBD range was 0.39–400 ng/ml, and CBD-glucuronide range was 0.78–200 ng/ml. There was no carryover and no matrix interferences. Accuracy in the study sample batch was within the ±15% acceptance criterion and imprecision was <15%.

### Behavior

Behavior tests occurred in controlled light, temperature, humidity, silent, pathogen-free environment. Mice were tested on the following schedule: open field test at 6 weeks of age, y maze test at 7 weeks of age, light dark box test at 8 weeks of age, puzzle box test for problem-solving behaviors at 9 weeks of age, elevated zero maze test for anxiety-like behaviors at 10 weeks of age, marble burying for compulsivity at 13 weeks of age, and Hargreaves for thermal pain sensitivity at 11 weeks of age. Open field, light/dark box, elevated zero maze, and puzzle box data were collected and analyzed using the Ethovision XT software from Noldus using version 8.5.

### Hargreaves test

The Hargreaves test is used to test murine thermal sensitivity [33]. We placed each mouse in a glass bottom enclosure heated to 30 °C/86 °F temperature to minimize errors arising from heat sink effects. Mice were habituated to the apparatus for 1 h the day before the test, and at least 30 min on the day of the test. The intensity of the heat source was set to 10 amps which produced withdraw latencies of 5–15 s in naïve animals. We tested eighteen CBD-exposed and twenty-one vehicle exposed female mice from seven different litters per exposure and eight CBD-exposed and nine vehicle exposed male mice from three different litters per exposure.

### Estrus cycle tracking

The vaginal cytology method was used to track the estrus cycle of female mice [55]. Immediately after the Hargreaves test, female mice were lavaged to collect cells from the vaginal wall. PBS was pipetted up and down 3 times within the vagina to obtain cells. Cells were mounted on a dry slide, overlaid with a coverslip, and immediately viewed at 200X magnification under bright field illumination. Estrus stage was determined based on the presence or absence of leukocytes, cornified epithelial, and nucleated epithelial cells [56].

### Open field test

The open field test measures murine anxiety-like behaviors and locomotor activity. We place the mice in a 44Wx44Lx25H cm arena under bright lighting conditions (900-1000lux) for 10-min sessions each. Ethovision tracking system measures total distance traveled in centimeters along with time spent in the outer and central zones of the box.

### Light dark box

The light dark box tests murine unconditioned anxiety-like behaviors [33]. The mice were placed in a box (45WX22.5LX28H cm) with one dark, covered section and one lit, open section, separated with a dark wall containing a door. Each mouse was placed into the closed section of the box for 5 min, then the door was removed, and the mouse was able to explore the open area under video monitoring for 5 min. We quantified time spent in the open and closed areas, and number of transitions between the two areas.

### Elevated zero maze

The elevated zero maze tests murine anxiety-like behaviors. Mice were placed individually on a circular runway (50 cm diameter, 5 cm wide track, 50 cm above ground) which is divided into four 90° quadrants. Two opposing quadrants are surrounded by 30 cm high walls while the in-between quadrants have no walls. The mice were placed facing the entrance of one of the walled quadrants. Time spent in each quadrant was video recorded and scored for the number of zone transitions, distance moved, and percentage of time in open and closed zones. Each mouse is tested for ten minutes.

### Y maze test

The y maze spontaneous alternation test quantifies murine spatial cognition. Mice were placed in the center of a y-shaped maze with three opaque arms at 120° angles from each other. The mouse was free to explore all three arms of the maze. Mice were monitored for either 10 min or 22 arm-changes, or whichever happened first. We calculate the percentage of “correct” and “incorrect” movements, where correct patterns are three subsequent arm changes (e.g., arm A to arm B to arm C) and incorrect patterns are three arm changes in repeated arms (e.g., arm A to arm B to arm A). Entry to the arm is marked once all four limbs have entered that arm.

### Marble burying test

Marble burying measures compulsivity in mice. Our apparatus is an 11 cm × 11 cm box filled with a layer of bedding and a 3 × 3 square of evenly placed blue marbles on top of the bedding under recording on the Ethovision video monitoring system. Mice were placed in the apparatus for 10 min. We quantified the total distance traveled and velocity of the mice from the Ethovision tracking system, and marble burying was quantified manually. We quantified the number of marbles buried, number of marbles re-buried, and time spent burying (seconds and percentage of total time).

### Puzzle box

The puzzle box measures murine cognition and problem-solving behaviors. The puzzle box is a Plexiglas white box divided by a removable barrier into two compartments: a brightly lit start zone (58 cm long, 28 cm wide) and a smaller covered goal zone (15 cm long, 28 cm wide). Mice are motivated to move into the goal zone by their aversion to the bright light in the start zone. We placed individual mice into the start zone and measured the time to move through the 4 cm wide underpass to the goal zone (dark compartment). Each mouse underwent nine trials (T1-T9) over the course of three days, with three trials each day. Each day the underpass was obstructed with increasing difficulty. For T1 (training) the underpass is clear, and the barrier has an open door over the location. On T2 and T3 (day 1) and T4 (day 2), the mice go through an underpass. On T5 and T6 (day 2) and T7 (day 3), the mice must dig through the sawdust-filled underpass to reach the goal zone. During T8 and T9 (day 3), the mice must remove a 4x4cm covering and then dig through sawdust to reach the goal zone. This sequence allows assessment of problem-solving abilities (T2, T5 and T8), learning/short-term memory (T3, T6, and T9), and repetition on the next day provides a measure of long-term memory (T4 and T7). We tested twelve vehicle-exposed female offspring from five litters, twelve CBD-exposed female offspring from five litters, twelve vehicle-exposed male offspring from four litters, and twelve CBD-exposed male offspring from four litters in the puzzle box.

### Preparation of acute prefrontal cortex (PFC) slices

Acute coronal PFC slices were obtained from P14 to 22 C57BL/6 male and female wild-type mice prenatally exposed to either CBD or vehicle in accordance with the Institutional Animal Care and Use Committees of the University of Colorado on Anschutz Medical Campus and National Institutes of Health guidelines. Mice were anesthetized with isoflurane and euthanized by decapitation. Immediately after decapitation, the brain was extracted and placed in icy cutting solution containing 215 mM sucrose, 20 mM glucose, 26 mM NaHCO_3_, 4 mM MgCl_2_, 4 mM MgSO_4_, 1.6 mM NaH_2_PO_4_, 1 mM CaCl_2_, and 2.5 mM KCl. Using a Leica VT1000S vibratome, the PFC was sectioned into 300μm thick slices. PFC slices were incubated at 32 °C for 30 min in 50% cutting solution and 50% artificial cerebrospinal fluid (ACSF) composed of 124 mM NaCl, 26 mM NaHCO_3_, 10 mM glucose, 2.5 mM KCl, 1 mM NaH_2_PO_4_, 2.5 mM CaCl_2_, and 1.3 mM MgSO_4_. After 30 min, this solution was replaced with ACSF at room temperature. For all two-photon and electrophysiology experiments, the slices were placed in a recording chamber and bathed in carbogenated (95% O_2_ / 5% CO_2_) ACSF at 30 °C and allowed to equilibrate for at least 30 min prior to the start of experiments.

### Two-photon Imaging

Two-photon imaging was performed on layer 2/3 pyramidal neurons at depths of 20–50 μm of PFC slices at P14-P22 using a two-photon microscope (Bruker) with a pulsed Ti:sapphire laser (MaiTai HP, Spectra Physics) tuned to 920 nm (4–5 mW at the sample). All experiments were controlled using the Prairie View (Bruker) software. Neurons were imaged at 30 °C in recirculating ACSF with 2 mM CaCl_2_, 1 mM MgCl_2_ aerated with 95% O_2_/5% CO_2_. For visualization, cells were whole-cell patched and filled with Alexa 488. For each neuron, image stacks (512 ×512 pixel; 0.047 um/pixel) with 1-μm z-steps were collected from secondary or tertiary distal apical dendrites. All images shown are maximal projections of three-dimensional image stacks after applying a median filter (2 ×2) to the raw image data. All protrusions on the dendritic shaft were counted as dendritic spines in images of green (Alexa 488) channel using ImageJ software (NIH). Dendritic spines density was calculated by dividing the number of spines by the dendritic length (in μm). Spine size was estimated from background-subtracted and bleed-through-corrected integrated pixel fluorescence intensity of the region of interest (~1 μm^2^) surrounding the spine head. This measurement was normalized to the mean fluorescence intensity of the dendritic segment adjacent to the dendritic spine of interest [37, 57]. Spine length/head width ratio was defined as the ratio of the length from the tip of the spine head to the base of spine neck (spine length) to the width across the spine head at its widest point (head width) [57].

### Electrophysiology and Two-photon glutamate uncaging

PFC layer 2/3 pyramidal neurons were identified by morphology and patched in the whole-cell (4–8 MΩ electrode resistance; 20–40 MΩ series resistance) current clamp configuration (MultiClamp 700B, Molecular Devices) within 40 μm of the slice surface. Using a potassium-based internal solution (136mM K-gluconate, 10 mM HEPES, 17.5 mM KCl, 9 mM NaCl, 1 mM MgCl_2_, 4 mM Na_2_-ATP, 0.4 mM Na-GTP, 0.2 mM Alexa 488, and ~300 mOsm, ~pH 7.26), spiking properties of layer 2/3 neurons were examined at 30 °C in recirculating ACSF with 2 mM CaCl_2_, 1 mM MgCl_2_. Excitability was measured by injection of depolarizing current steps (50–250 pA, 300 ms). Resting membrane potential was recorded prior to the first depolarizing current step. Minimum current required to elicit an action potential was defined as the smallest current step that triggered at least one spike. The spike threshold was defined as the potential at which the spike is triggered. The change in potential (∆Vm) was defined as the difference (absolute value) between the resting membrane potential and spike threshold. For two-photon glutamate uncaging experiments, whole-cell recordings in the voltage clamp configuration (V_hold_ = −65 mV) were performed in 1 μM TTX and 2.5 mM MNI-glutamate (Tocris) containing ACSF at 30 °C. Using cesium-based internal solution (135 mM Cs-methanesulfonate, 10 mM HEPES, 10 mM Na_2_ phosphocreatine, 4 mM MgCl_2_, 4 mM Na_2_-ATP, 0.4 mM Na-GTP, 3 mM Na L-ascorbate, 0.2 mM Alexa 488, and ~300 mOsm, ~pH 7.25), individual dendritic spines (on secondary or tertiary apical dendritic branches, 50–100 μm from soma) were targeted by two-photon glutamate uncaging and uncaging evoked excitatory postsynaptic currents (uEPSCs) were recorded [37]. uEPSC amplitudes were quantified as the average (5–8 test pulses at 0.1 Hz) from a 1 ms window centered on the maximum current amplitude within 30 ms after uncaging pulse delivery.

### Statistical analyses

Unless otherwise specified, data were collected and segregated by sex, exposure group, and genotype. We completed a D’Agostino and Pearson test to determine if data were normally distributed. When normally distributed, data within each direct comparison (two exposure groups within one sex and one genotype) were analyzed via a t test. If data were nonnormally distributed, we completed a Wilcoxon rank sum test within each direct comparison. P-values less than 0.05 are reported as significant.

### Supplementary information

Supplemental Table 1

Supplemental Figure 2

Supplemental Figure 1

## References

[1] Wibowo N, Purwosunu Y, Sekizawa A, Farina A, Tambunan V, Bardosono S (2012). Vitamin B_6_ supplementation in pregnant women with nausea and vomiting. Int J Gynaecol Obstet.

[2] Volkow ND, Han B, Compton WM, McCance-Katz EF (2019). Self-reported medical and nonmedical cannabis use among pregnant women in the United States. JAMA.

[3] Metz TD, Silver RM, McMillin GA, Allshouse AA, Jensen TL, Mansfield C (2019). Prenatal marijuana use by self-report and umbilical cord sampling in a state with marijuana legalization. Obstet Gynecol.

[4] Bidwell CL, YorkWilliams SL, Mueller RL, Bryan AD, Hutchison KE (2018). Exploring cannabis concentrates on the legal market: user profiles, product strength, and health-related outcomes. Addict Behav Rep.

[5] Parker LA, Rock EM, Limebeer CL (2011). Regulation of nausea and vomiting by cannabinoids. Br J Pharmacol.

[6] Rock EM, Limebeer CL, Pertwee RG, Mechoulam R, Parker LA (2021). Therapeutic potential of cannabidiol, cannabidiolic acid, and cannabidiolic acid methyl ester as treatments for nausea and vomiting. Cannabis Cannabinoid Res.

[7] Rock E, Bolognini D, Limebeer C, Cascio M, Anavi-Goffer S, Fletcher P (2012). Cannabidiol, a non-psychotropic component of cannabis, attenuates vomiting and nausea-like behaviour via indirect agonism of 5-HT1A somatodendritic autoreceptors in the dorsal raphe nucleus. Brit J Pharmacol.

[8] Abernethy A. Hemp Production and the 2018 Farm Bill - 07/25/2019. FDA; 2019. https://www.fda.gov/news-events/congressional-testimony/hemp-production-and-2018-farm-bill-07252019. Accessed 22 Nov 2022.

[9] Ochiai W, Kitaoka S, Kawamura T, Hatogai J, Harada S, Iizuka M (2021). Maternal and fetal pharmacokinetic analysis of cannabidiol during pregnancy in mice. Drug Metab Dispos.

[10] Grandl J, Kim SE, Uzzell V, Bursulaya B, Petrus M, Bandell M (2010). Temperature-induced opening of TRPV1 ion channel is stabilized by the pore domain. Nat Neurosci.

[11] Martínez-Aguirre C, Carmona-Cruz F, Velasco AL, Velasco F, Aguado-Carrillo G, Cuéllar-Herrera M, et al. Cannabidiol acts at 5-HT1A receptors in the human brain: relevance for treating temporal lobe epilepsy. Front Behav Neurosci. 2020;14. https://www.ncbi.nlm.nih.gov/pmc/articles/PMC7770178/. 10.3389/fnbeh.2020.611278 PMC7770178 33384591

[12] Anand U, Jones B, Korchev Y, Bloom SR, Pacchetti B, Anand P (2020). CBD effects on TRPV1 signaling pathways in cultured DRG neurons. J Pain Res.

[13] Zhang H-XB, Heckman L, Niday Z, Jo S, Fujita A, Shim J, et al. Cannabidiol activates neuronal Kv7 channels. eLife. 2022;11:e73246. 10.7554/eLife.73246. 10.7554/eLife.73246 PMC8856652 35179483

[14] Hutson MR, Keyte AL, Hernández-Morales M, Gibbs E, Kupchinsky ZA, Argyridis I (2017). Temperature-activated ion channels in neural crest cells confer maternal fever-associated birth defects. Sci Signal.

[15] Terzian ALB, Aguiar DC, Guimarães FS, Moreira FA (2009). Modulation of anxiety-like behaviour by transient receptor potential vanilloid type 1 (TRPV1) channels located in the dorsolateral periaqueductal gray. Eur Neuropsychopharmacol.

[16] Hurtado-Zavala JI, Ramachandran B, Ahmed S, Halder R, Bolleyer C, Awasthi A (2017). TRPV1 regulates excitatory innervation of OLM neurons in the hippocampus. Nat Commun.

[17] Cavanaugh DJ, Chesler AT, Jackson AC, Sigal YM, Yamanaka H, Grant R (2011). Trpv1 reporter mice reveal highly restricted brain distribution and functional expression in arteriolar smooth muscle cells. J. Neurosci..

[18] Mezey É, Tóth ZE, Cortright DN, Arzubi MK, Krause JE, Elde R (2000). Distribution of MRNA for vanilloid receptor subtype 1 (VR1), and VR1-like Immunoreactivity, in the central nervous system of the rat and human. Proc Natl Acad Sci.

[19] The limbic system, 2022. https://qbi.uq.edu.au/brain/brain-anatomy/limbic-system. Accessed 22 Nov 2022.

[20] Rompala G, Nomura Y, Hurd YL (2021). Maternal cannabis use is associated with suppression of immune gene networks in placenta and increased anxiety phenotypes in offspring. Proc Natl Acad Sci.

[21] Lu H, Liu Q (2017). Serotonin in the frontal cortex: a potential therapeutic target for neurological disorders. Biochem Pharmacol.

[22] Bert B, Fink H, Rothe J, Walstab J, Bönisch H (2008). Learning and memory in 5-HT1A-receptor mutant mice. Behav Brain Res.

[23] Hanswijk SI, Spoelder M, Shan L, Verheij MMM, Muilwijk OG, Li W (2020). Gestational factors throughout fetal neurodevelopment: the serotonin link. Int J Mol Sci.

[24] Riedel WJ, Klaassen T, Deutz NE, van Someren A, van Praag HM (1999). Tryptophan depletion in normal volunteers produces selective impairment in memory consolidation. Psychopharmacology.

[25] Del’Guidice T, Lemay F, Lemasson M, Levasseur-Moreau J, Manta S, Etievant A (2014). Stimulation of 5-HT2C receptors improves cognitive deficits induced by human tryptophan hydroxylase 2 loss of function mutation. Neuropsychopharmacology.

[26] Bonnin A, Peng W, Hewlett W, Levitt P (2006). Expression mapping of 5-HT1 serotonin receptor subtypes during fetal and early postnatal mouse forebrain development. Neuroscience.

[27] Bar-Peled O, Gross-Isseroff R, Ben-Hur H, Hoskins I, Groner Y, Biegon A (1991). Fetal human brain exhibits a prenatal peak in the density of serotonin 5-HT1A receptors. Neurosci Lett.

[28] Dirkx N, Miceli F, Taglialatela M, Weckhuysen S. The role of Kv7.2 in neurodevelopment: insights and gaps in our understanding. Front Physiol. 2020;11. https://www.ncbi.nlm.nih.gov/pmc/articles/PMC7657400/. 10.3389/fphys.2020.570588 PMC7657400 33192566

[29] Miceli F, Soldovieri MV, Ambrosino P, De Maria M, Migliore M, Migliore R (2015). Early-onset epileptic encephalopathy caused by gain-of-function mutations in the voltage sensor of kv7.2 and kv7.3 potassium channel subunits. J Neurosci.

[30] Fleckenstein J, Sittl R, Averbeck B, Lang PM, Irnich D, Carr RW (2013). Activation of axonal Kv7 channels in human peripheral nerve by flupirtine but not placebo - therapeutic potential for peripheral neuropathies: results of a randomised controlled trial. J Transl Med.

[31] Zhou X, Song M, Chen D, Wei L, Yu SP (2011). Potential role of KCNQ/M-channels in regulating neuronal differentiation in mouse hippocampal and embryonic stem cell-derived neuronal cultures. Exp Neurol.

[32] The 2020 CBD survey. The Checkup. 2020. https://www.singlecare.com/blog/cbd-survey/. Accessed 22 Nov 2022.

[33] Cheah M, Fawcett JW, Andrews MR (2017). Assessment of thermal pain sensation in rats and mice using the hargreaves test. Bio Protoc.

[34] Vinogradova EP, Zhukov DA, Batuev AS (2003). The effects of stages of the estrous cycle on pain thresholds in female white rats. Neurosci Behav Physiol.

[35] Marsch R, Foeller E, Rammes G, Bunck M, Kössl M, Holsboer F (2007). Reduced anxiety, conditioned fear, and hippocampal long-term potentiation in transient receptor potential vanilloid type 1 receptor-deficient mice. J Neurosci.

[36] Ben Abdallah NM-B, Fuss J, Trusel M, Galsworthy MJ, Bobsin K, Colacicco G (2011). The puzzle box as a simple and efficient behavioral test for exploring impairments of general cognition and executive functions in mouse models of schizophrenia. Exp Neurol.

[37] Oh WC, Lutzu S, Castillo PE, Kwon H-B (2016). De novo synaptogenesis induced by GABA in the developing mouse cortex. Science.

[38] Kwon H-B, Sabatini BL (2011). Glutamate induces de novo growth of functional spines in developing cortex. Nature.

[39] Oh WC, Hill TC, Zito K (2013). Synapse-specific and size-dependent mechanisms of spine structural plasticity accompanying synaptic weakening. Proc Natl Acad Sci USA.

[40] Kleinjan MS, Buchta WC, Ogelman R, Hwang I-W, Kuwajima M, Hubbard DD, et al. Dually innervated dendritic spines develop in the absence of excitatory activity and resist plasticity through tonic inhibitory crosstalk. Neuron. 2022. 10.1016/j.neuron.2022.11.002. 10.1016/j.neuron.2022.11.002 PMC9899020 36395772

[41] Yao J, Liu B, Qin F (2011). Modular thermal sensors in temperature-gated transient receptor potential (TRP) channels. Proc Natl Acad Sci.

[42] Muller C, Morales P, Reggio PH. Cannabinoid ligands targeting TRP channels. Front Mol Neurosci. 2019;11. https://pubmed.ncbi.nlm.nih.gov/30697147/. 10.3389/fnmol.2018.00487 PMC6340993 30697147

[43] Xu S, Cheng Y, Keast JR, Osborne PB (2008). 17β-estradiol activates estrogen receptor β-signalling and inhibits transient receptor potential vanilloid receptor 1 activation by capsaicin in adult rat nociceptor neurons. Endocrinology.

[44] Payrits M, Sághy É, Csekő K, Pohóczky K, Bölcskei K, Ernszt D (2017). Estradiol sensitizes the transient receptor potential vanilloid 1 receptor in pain responses. Endocrinology.

[45] Morecraft RJ, Yeterian EH. Prefrontal cortex. In: Ramachandran VS, editor. Encyclopedia of the human brain. New York: Academic Press; 2002, pp 11–26. 10.1016/B0-12-227210-2/00285-5.

[46] Albert PR, Vahid-Ansari F, Luckhart C. Serotonin-prefrontal cortical circuitry in anxiety and depression phenotypes: pivotal role of pre- and post-synaptic 5-HT1A receptor expression. Front Behav Neurosci. 2014;8. https://pubmed.ncbi.nlm.nih.gov/24936175/10.3389/fnbeh.2014.00199 PMC4047678 24936175

[47] Peng H, Bian X, Ma F, Wang K-W (2017). Pharmacological modulation of the voltage-gated neuronal Kv7/KCNQ/M-channel alters the intrinsic excitability and synaptic responses of pyramidal neurons in rat prefrontal cortex slices. Acta Pharmacol Sin.

[48] Wanner NM, Colwell M, Drown C, Faulk C (2021). Developmental cannabidiol exposure increases anxiety and modifies genome-wide brain DNA methylation in adult female mice. Clin Epigenetics.

[49] Marchand G, Masoud AT, Govindan M, Ware K, King A, Ruther S (2022). Birth outcomes of neonates exposed to marijuana in utero: a systematic review and meta-analysis. JAMA Network Open.

[50] Pennypacker SD, Cunnane K, Cash MC, Romero-Sandoval EA (2022). Potency and therapeutic THC and CBD ratios: U.S. cannabis markets overshoot. Front Pharmacol.

[51] Deiana S, Watanabe A, Yamasaki Y, Amada N, Arthur M, Fleming S (2012). Plasma and Brain Pharmacokinetic Profile of Cannabidiol (CBD), Cannabidivarine (CBDV), Δ^9^-Tetrahydrocannabivarin (THCV) and Cannabigerol (CBG) in rats and mice following oral and intraperitoneal administration and CBD action on obsessive-compulsive behaviour. Psychopharmacology.

[52] Millar SA, Stone NL, Yates AS, O’Sullivan SE (2018). A systematic review on the pharmacokinetics of cannabidiol in humans. Front Pharmacol.

[53] Ewing LE, Skinner CM, Quick CM, Kennon-McGill S, McGill MR, Walker LA (2019). Hepatotoxicity of a cannabidiol-rich cannabis extract in the mouse model. Molecules.

[54] Sempio C, Huestis MA, Kaplan B, Klawitter J, Christians U, Henthorn TK (2022). Urinary clearance of 11-nor-9-carboxy-Δ9 -tetrahydrocannabinol: a detailed pharmacokinetic analysis. Drug Test Anal.

[55] Byers SL, Wiles MV, Dunn SL, Taft RA (2012). Mouse estrous cycle identification tool and images. PLOS One.

[56] Felicio LS, Nelson JF, Finch CE (1984). Longitudinal studies of estrous cyclicity in aging C57BL/6J mice: II. Cessation of cyclicity and the duration of persistent vaginal cornification1. Biol Reprod.

[57] Woods GF, Oh WC, Boudewyn LC, Mikula SK, Zito K (2011). Loss of PSD-95 enrichment is not a prerequisite for spine retraction. J Neurosci.

