# Supplementary material for: Fetal cannabidiol (CBD) exposure alters thermal pain sensitivity, problem-solving, and prefrontal cortex excitability
Source: Mol Psychiatry. 2023 Jul 11;28(8):3397–413. doi: 10.1038/s41380-023-02130-y (PMC10618089; doi:10.1038/s41380-023-02130-y)
Supplement: Supplementary file 1 — Supplemental Table 1 [file 41380_2023_2130_MOESM1_ESM.pdf]

Table 1. Data for anxiety-like behavior

Test	Measure	Female Vehicle			Female CBD			Female Effect Size	Female vehicle to female CBD	Distrobution	Test used
		N pups	N litters	Value ± SEM	N pups	N litters	Value ± SEM	Vehicle - CBD Mean	P value		
Open field test	Freuqnecy in center zone	13	5	49.93 ± 3.83	25	7	50.12 ± 2.29	-0.19	0.965	Normal	T-Test
Open field test	Velocity (cm/s)	13	5	11.34 ± 0.92	25	7	10.88 ± 0.51	0.46	0.525	Nonnormal	Wilcoxin rank sum test
Open field test	Total distance moved (cm)	13	5	932.47 ± 81.25	25	7	925.19 ± 50.00	7.28	0.936	Normal	T-Test
Open field test	Time in center zone (s)	13	5	90.20 ± 11.05	25	7	89.19 ± 6.36	1.01	0.933	Normal	T-Test
Open field test	Time moving (s)	13	5	70.46 ± 7.65	25	7	71.90 ± 4.62	-1.44	0.865	Normal	T-Test
Open field test	Time still (s)	13	5	19.72 ± 3.82	25	7	17.28 ± 2.10	2.44	0.225	Nonnormal	Wilcoxin rank sum test
Light dark box	Total distance moved in open area (cm)	6	3	740.45 ± 106.50	21	5	1033.55 ± 92.01	-293.1	0.071	Nonnormal	Wilcoxin rank sum test
Light dark box	Mean velocity (cm/s)	6	3	6.79 ± 0.30	21	5	6.83 ± 0.29	-0.04	0.572	Nonnormal	Wilcoxin rank sum test
Light dark box	Duration in open area (s)	6	3	263.09 ± 17.52	21	5	234.12 ± 11.89	28.97	0.225	Nonnormal	Wilcoxin rank sum test
Light dark box	Duration in near zone (s)	6	3	219.84 ± 16.48	21	5	159.26 ± 15.01	60.58	0.052	Normal	T-Test
Light dark box	Duration in middle zone (s)	6	3	17.34 ± 3.37	21	5	27.23 ± 2.54	-9.89	0.082	Normal	T-Test
Light dark box	Duration in far zone (s)	6	3	25. 28 ± 5.92	21	5	47.62 ± 11.22	-22.34	0.306	Nonnormal	Wilcoxin rank sum test
Light dark box	Latency to enter near zone (s)	6	3	1.49 ± 0.82	21	5	6.90 ± 4.79	-5.41	0.978	Nonnormal	Wilcoxin rank sum test
Light dark box	Latency to enter middle zone (s)	6	3	2.15 ± 1.17	21	5	18.85 ± 6.12	-16.7	0.112	Nonnormal	Wilcoxin rank sum test
Light dark box	Latency to enter far zone (s)	6	3	10.488 ± 5.497	21	5	20.30 ± 6.25	-9.812	0.428	Nonnormal	Wilcoxin rank sum test
Light dark box	Frequency in near zone	6	3	19.83 ± 3.84	21	5	27.33 ± 2.87	-7.5	0.207	Normal	T-Test
Light dark box	Frequency in middle zone	6	3	5.59 ± 2.28	21	5	20.33 ± 1.97	-14.74	0.184	Normal	T-Test
Light dark box	Frequency in far zone	6	3	7.00 ± 1.39	21	5	10.95 ± 1.55	-3.95	0.124	Nonnormal	Wilcoxin rank sum test
Elevated zero maze	Velocity (cm/s)	13	5	5.27 ± 0.36	25	7	5.25 ± 0.26	0.02	0.969	Normal	T-Test
Elevated zero maze	Frequency in light area	13	5	33.85 ± 3.35	25	7	31.68 ± 2.17	2.17	0.577	Normal	T-Test
Elevated zero maze	Total distance moved (cm)	13	5	1573.94 ± 108.78	25	7	1570.05 ± 77.47	3.89	0.976	Normal	T-Test
Elevated zero maze	Time in closed area (s)	13	5	212.67 ± 9.16	25	7	206.69 ± 6.93	5.98	0.977	Nonnormal.	Wilcoxin rank sum test
Elevated zero maze	Time in open area (s)	13	5	87.43 ± 9.16	25	7	93.41 ± 6.93	-5.98	0.977	Nonnormal	Wilcoxin rank sum test
Elevated zero maze	Frequncy in closed area	13	5	34 ± 3.34	25	7	31.6 ± 2.14	2.4	0.534	Normal	T-Test

Test	Measure	Male Vehicle			Male CBD			Male Effect Size	Male vehicle to Male CBD	Distrobution	Test used
		N pups	N litters	Value ± SEM	N pups	N litters	Value ± SEM	Vehilce - CBD Mean	P value		
Open field test	Freuqnecy in center zone	19	6	49.26 ± 4.78	23	7	48.87 ± 2.88	0.39	0.942	Normal	T-Test
Open field test	Velocity (cm/s)	19	6	10.69 ± 0.63	23	7	10.71 ± 0.54	-0.02	0.978	Nonnormal	Wilcoxin rank sum test
Open field test	Total distance moved (cm)	19	6	913.97 ± 90.52	23	7	930.76 ± 53.62	-16.79	0.869	Normal	T-Test
Open field test	Time in center zone (s)	19	6	89.40 ± 10.05	23	7	90.76 ± 6.00	-1.36	0.904	Normal	T-Test
Open field test	Time moving (s)	19	6	70.76 ± 6.95	23	7	72.53 ± 4.07	-1.77	0.821	Normal	T-Test
Open field test	Time still (s)	19	6	18.63 ± 3.72	23	7	18.23 ± 2.35	0.4	0.551	Nonnormal	Wilcoxin rank sum test
Light dark box	Total distance moved in open area (cm)	13	4	854.22 ± 89.37	24	6	1191.09 ± 165.01	-336.87	0.105	Nonnormal	Wilcoxin rank sum test
Light dark box	Mean velocity (cm/s)	13	4	7.12 ± 0.23	24	6	6.99 ± 0.50	0.13	0.484	Nonnormal	Wilcoxin rank sum test
Light dark box	Duration in open area (s)	13	4	233.82 ± 18.18	24	6	246.27 ± 14.30	-12.45	0.239	Nonnormal	Wilcoxin rank sum test
Light dark box	Duration in near zone (s)	13	4	180.42 ± 19.22	24	6	179.64 ± 13.14	0.78	0.973	Normal	T-Test
Light dark box	Duration in middle zone (s)	13	4	21.65 ± 2.99	24	6	31.87 ± 2.38	-10.22	0.014	Normal	T-Test
Light dark box	Duration in far zone (s)	13	4	31.75 ± 3.49	24	6	37.65 ± 2.78	-5.9	0.156	Nonnormal	Wilcoxin rank sum test
Light dark box	Latency to enter near zone (s)	13	4	3.57 ± 2.82	24	6	3.61 ± 1.68	-0.04	0.766	Nonnormal	Wilcoxin rank sum test
Light dark box	Latency to enter middle zone (s)	13	4	12.98 ± 4.98	24	6	13.35 ± 4.37	-0.37	0.915	Nonnormal	Wilcoxin rank sum test
Light dark box	Latency to enter far zone (s)	13	4	16.19 ± 5.65	24	6	15.85 ± 4.60	0.34	0.965	Nonnormal	Wilcoxin rank sum test
Light dark box	Frequency in near zone	13	4	21.23 ± 2.52	24	6	24.79 ± 2.67	-3.56	0.391	Normal	T-Test
Light dark box	Frequency in middle zone	13	4	18.62 ± 1.95	24	6	22.25 ± 1.72	-3.63	0.196	Normal	T-Test
Light dark box	Frequency in far zone	13	4	8.85 ± 0.89	24	6	10.54 ± 0.87	-1.69	0.193	Nonnormal	Wilcoxin rank sum test
Elevated zero maze	Velocity (cm/s)	23	7	4.79 ± 0.26	24	7	4.92 ± 0.26	-0.13	0.733	Normal	T-Test
Elevated zero maze	Frequency in light area	23	7	27.43 ± 2.15	24	7	27.04 ± 2.29	0.39	0.901	Normal	T-Test
Elevated zero maze	Total distance moved (cm)	23	7	1437.06 ± 78.82	24	7	1474.54 ± 79.04	-37.48	0.739	Normal	T-Test
Elevated zero maze	Time in closed area (s)	23	7	222.98 ± 6.21	24	7	218.35 ± 5.76	4.63	0.395	Nonnormal.	Wilcoxin rank sum test
Elevated zero maze	Time in open area (s)	23	7	77.12 ± 6.22	24	7	81.75 ± 5.76	-4.63	0.395	Nonnormal	Wilcoxin rank sum test
Elevated zero maze	Frequncy in closed area	23	7	27.30 ± 2.18	24	7	26.92 ± 2.32	0.38	0.904	Normal	T-Test
