# Supplementary figures and images for: Fetal cannabidiol (CBD) exposure alters thermal pain sensitivity, problem-solving, and prefrontal cortex excitability
Source: Mol Psychiatry. 2023 Jul 11;28(8):3397–413. doi: 10.1038/s41380-023-02130-y (PMC10618089; doi:10.1038/s41380-023-02130-y)

# Open field test

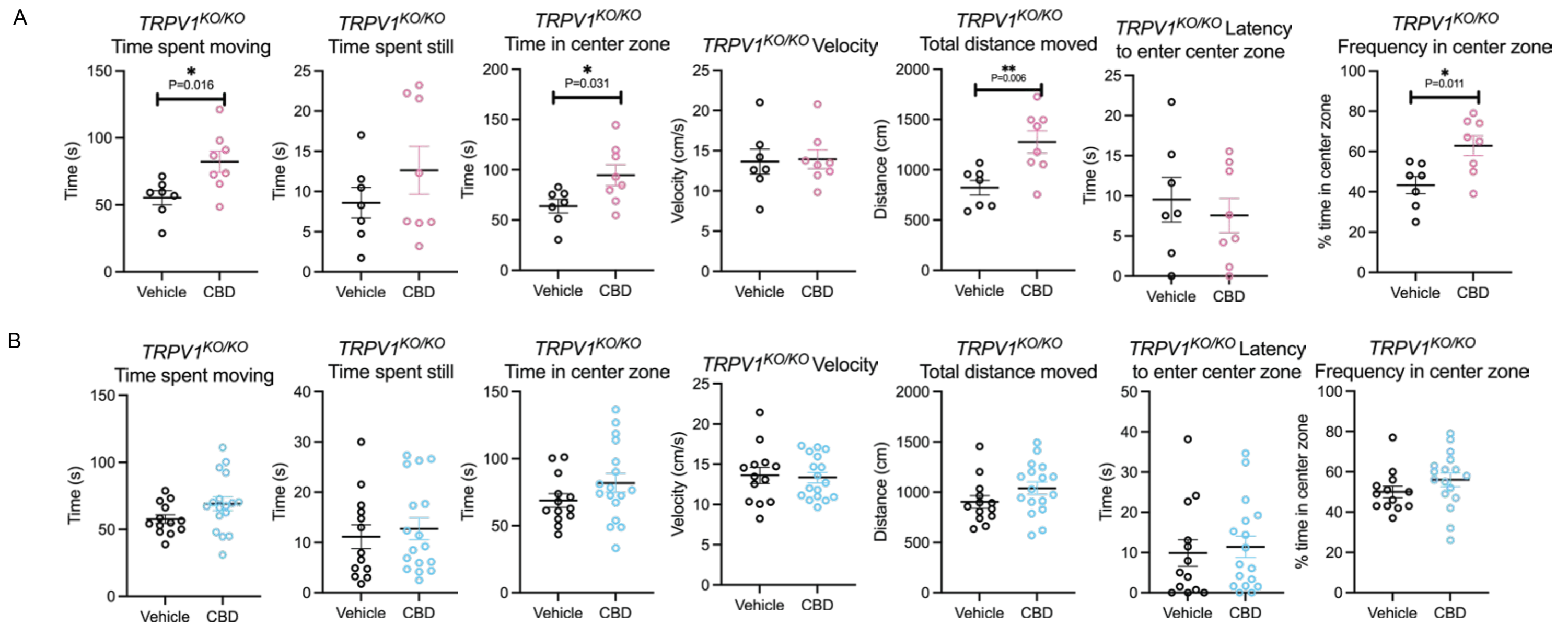

# Light dark box

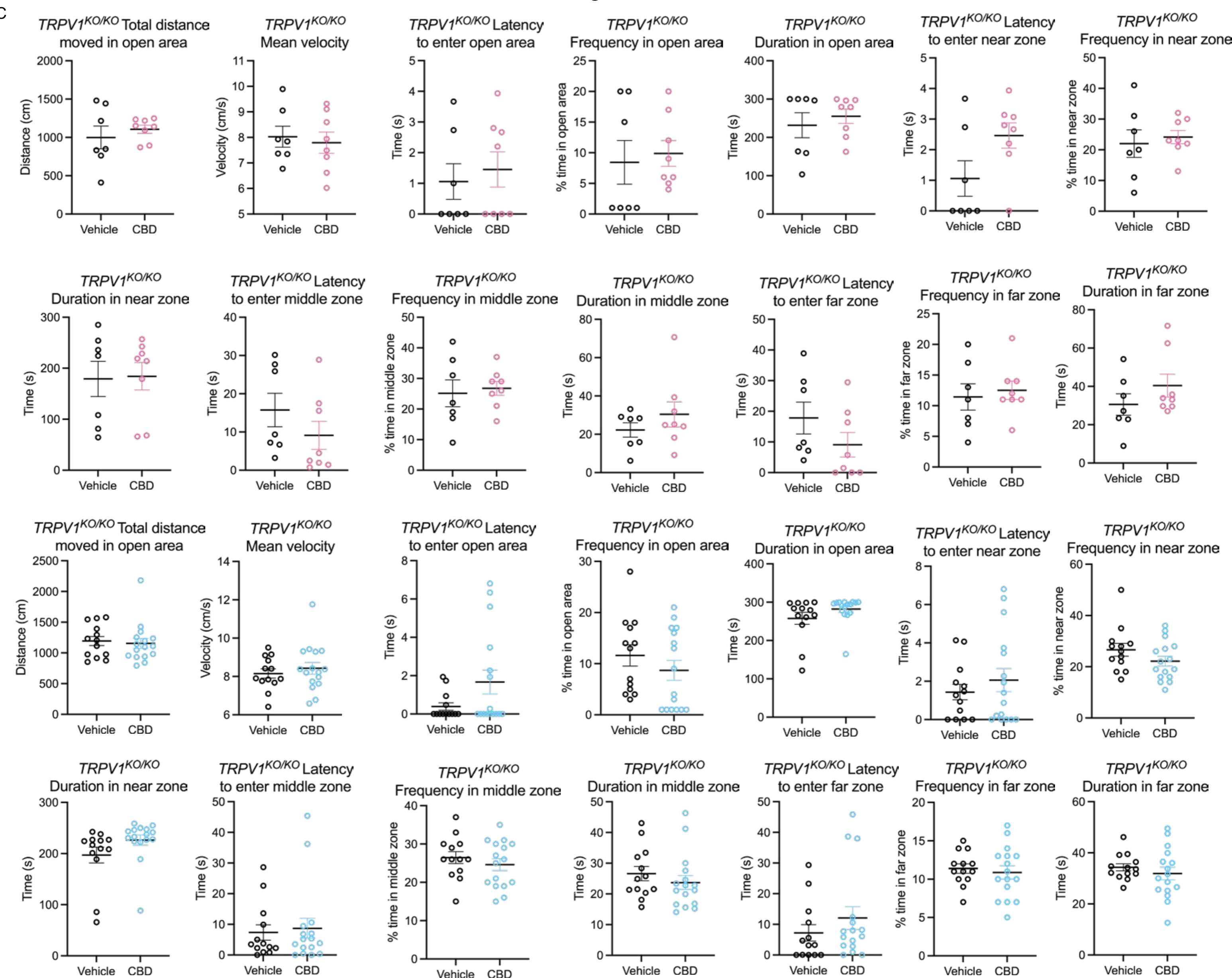

Supplement: Supplementary file 2 — Supplemental Figure 2 [file 41380_2023_2130_MOESM2_ESM.pdf]

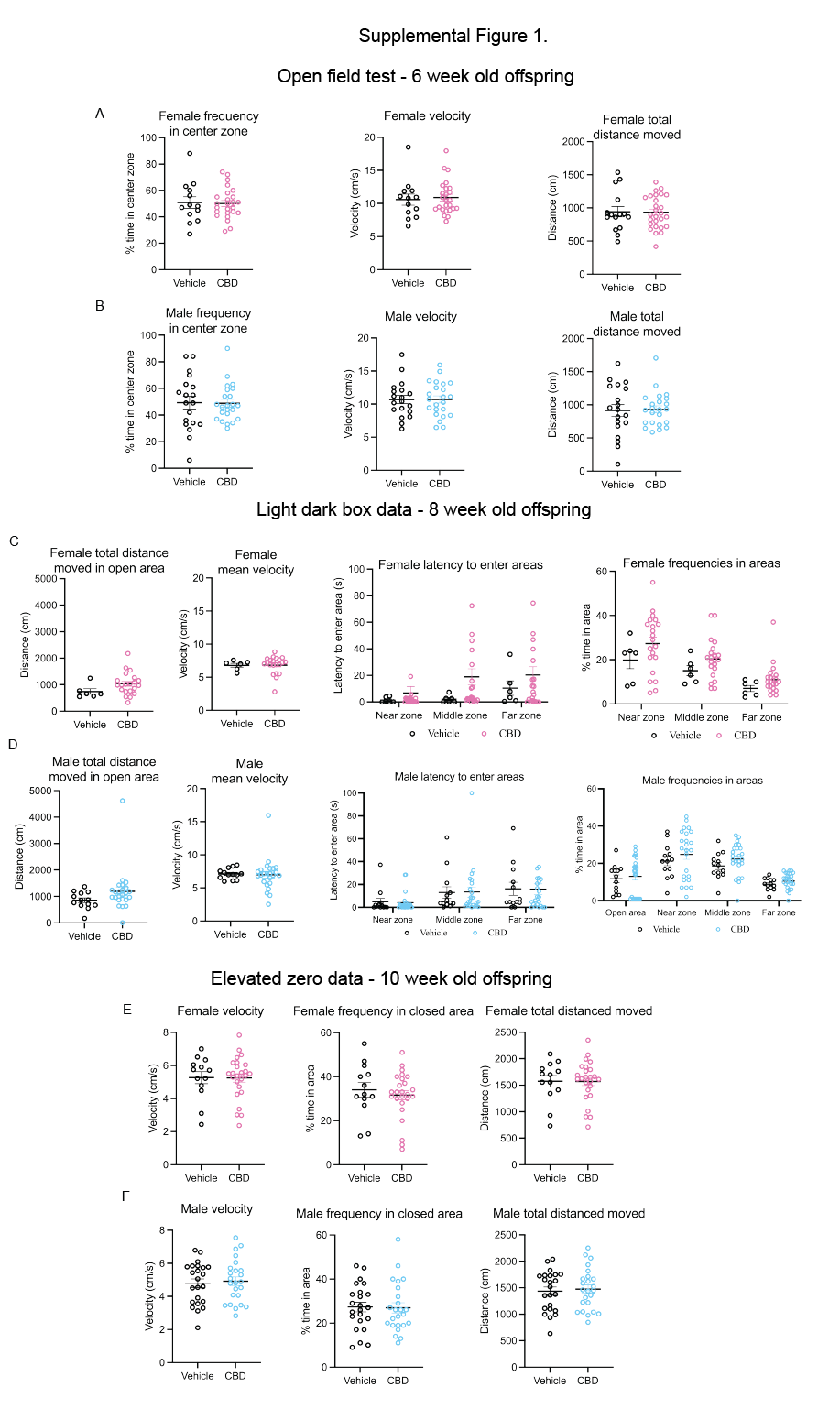

Supplement: Supplementary file 3 — Supplemental Figure 1 [file 41380_2023_2130_MOESM3_ESM.png]
